# Discovery of antimicrobial compounds targeting bacterial type FAD synthetases

**DOI:** 10.1080/14756366.2017.1411910

**Published:** 2017-12-19

**Authors:** María Sebastián, Ernesto Anoz-Carbonell, Begoña Gracia, Pilar Cossio, José Antonio Aínsa, Isaías Lans, Milagros Medina

**Affiliations:** a Departamento de Bioquímica y Biología Molecular y Celular, Facultad de Ciencias, Universidad de Zaragoza, Zaragoza, Spain;; b Institute of Biocomputation and Physics of Complex Systems (BIFI-IQFR and CBsC-CSIC Joint Units), Universidad de Zaragoza, Zaragoza, Spain;; c Grupo de Genética de Micobacterias, Departamento de Microbiología, Medicina Preventiva y Salud Pública. Facultad de Medicina, Universidad de Zaragoza, Zaragoza, Spain;; d CIBER Enfermedades Respiratorias (CIBERES), Instituto de Salud Carlos III, Madrid, Spain;; e Department of Theoretical Biophysics, Max Planck Institute of Biophysics, Frankfurt, Germany;; f Biophysics of Tropical Diseases, Max Planck Tandem Group, University of Antioquia, Medellín, Colombia

**Keywords:** Bacterial FAD Synthetase, high-throughput screening, *Streptococcus pneumoniae*, drug discovery

## Abstract

The increase of bacterial strains resistant to most of the available antibiotics shows a need to explore novel antibacterial targets to discover antimicrobial drugs. Bifunctional bacterial FAD synthetases (FADSs) synthesise the flavin mononucleotide (FMN) and flavin adenine dinucleotide (FAD). These cofactors act in vital processes as part of flavoproteins, making FADS an essential enzyme. Bacterial FADSs are potential antibacterial targets because of differences to mammalian enzymes, particularly at the FAD producing site. We have optimised an activity-based high throughput screening assay targeting *Corynebacterium ammoniagenes* FADS (*Ca*FADS) that identifies inhibitors of its different activities. We selected the three best high-performing inhibitors of the FMN:adenylyltransferase activity (FMNAT) and studied their inhibition mechanisms and binding properties. The specificity of the *Ca*FADS hits was evaluated by studying also their effect on the *Streptococcus pneumoniae* FADS activities, envisaging differences that can be used to discover species-specific antibacterial drugs. The antimicrobial effect of these compounds was also evaluated on *C. ammoniagenes*, *S. pneumoniae,* and *Mycobacterium tuberculosis* cultures, finding hits with favourable antimicrobial properties.

## Graphical Abstract

**Figure:**
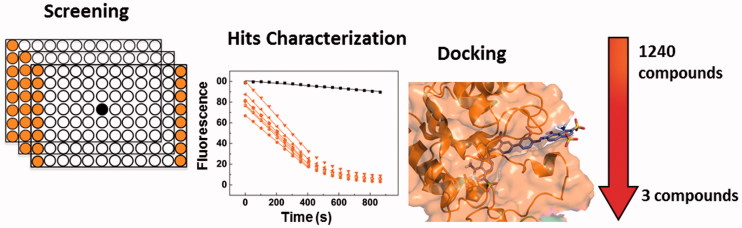

## Introduction

An important innovation gap in the discovering of antibiotics has occurred during the last two decades^1^, with only five new classes available and 51 new antimicrobials in clinical development^2–4^. In addition, the selection of multi-drug resistant microorganisms^5^ encourages to search for new antimicrobial drugs capable of inhibiting novel protein targets, such as those controlling the biosynthesis of essential biomolecules. Flavin mononucleotide (FMN) and flavin adenine dinucleotide (FAD) are the cofactors of flavoproteins. All living organisms contain a great number of such proteins and many of them are involved in essential functions^6–8^, including protein folding^9^, electron transport in the respiratory and photosynthetic chains^10^, β-oxidation of fatty acids^11^, nucleotide synthesis or signal transduction^12^, among others. Lack, or low levels, of FMN and FAD lead to the accumulation of apoflavoproteins, unable to perform the flavin-dependent functions, resulting in the concomitant death of the cell or the organism^13^^,^^14^. Prokaryotic bifunctional FAD synthetases (FADS) synthesise both FMN and FAD, being therefore potential new antimicrobial targets^15^. Such hypothesis is sustained by several facts; (i) halting the production of FMN and FAD prevents, from the very beginning, all pathways that involve flavoproteins and flavoenzymes, (ii) in most bacteria the only pathway for FMN and FAD biosynthesis occurs with bifunctional FADS^13^^,^^14^, (iii) prokaryotic FADSs differ structurally and biochemically from the mammalian proteins that transform FMN into FAD^16–19^, so drugs that target these proteins are likely to be selective for bacteria and (iv) the availability of structures of several bacterial FADSs facilitates the design of both inhibitory drugs and activity assays^20–22^.

Bacterial FADSs have both ATP:riboflavin kinase (RFK, EC 2.7.1.26) and ATP:FMN:adenylyltransferase (FMNAT, EC 2.7.7.2) activities, being the latter reversible (FAD pyrophosphorylase) in some species^17^^,^^23^. FADSs synthesise FMN and FAD from riboflavin (RF, vitamin B2) through two sequential reactions: RF is first phosphorylated to FMN by the RFK activity, and then the FMNAT activity transfers an adenylyl group from ATP to FMN producing FAD. These catalytic activities are performed by two almost independent modules (Supplementary Figure SD1). The C-terminus module produces FMN from RF (named RFK module), while the N-terminal module transforms FMN into FAD (FMNAT module). The RFK module shows sequence and structural homology with the monofunctional eukaryotic RFKs, while the FMNAT module does not present neither sequence nor structural similarity with the proteins that synthesise FAD in mammals^16^^,^^21^^,^^24^^,^^25^. Because the enzymes leading to FAD production in prokaryotes and eukaryotes use different chemistry, and belong to different structural families, potential inhibitors that specifically target the FMNAT module of bacterial FADSs are an interesting option for the novel drug development^15^.

In this work, we have used as a model the FADS from the non-pathogenic organism *Corynebacterium ammoniagenes* (*Ca*FADS), which is the best known model to characterise members of the prokaryotic FADSs family^17^^,^^24^^,^^26–30^, in an activity-based high-throughput screening (HTS) assay to find potential inhibitors. The HTS hits were assayed to determine their specificity and potency for the RFK and FMNAT activities. We also studied the kinetic inhibition mechanism of the three most potent and selective inhibitors of the FMNAT activity (FMNAT hits), as well as their binding properties. Furthermore, considering the structural similarity among *Ca*FADS and the FADSs from the human pathogens *Streptococcus pneumoniae* (included in the World Health Organisation [WHO] priority list of antibiotic resistant pathogens; *Spn*FADS) and *Mycobacterium tuberculosis* (the World’s leading infectious killer; *Mt*FADS), we explored the potential antimicrobial effect of the FADS HTS hits in these microorganisms by determining their minimal inhibitory concentration (MIC). Some of the HTS hits demonstrated high FADS inhibitory activity *in vitro*, but their antimicrobial activity revealed that uptake of these compounds by bacterial cells could be suboptimal. Collectively, our results validate our approach for discovering antimicrobials targeting bacterial FADSs, and for identifying inhibitors that constitute a great starting point for future developments of novel antimicrobials.

## Methods

### Protein purification and quantification

Recombinant *Ca*FADS was overexpressed in BL21 (DE3) *E. coli* cells and purified as previously described^30^. Recombinant *Spn*FADS was overexpressed in *E. coli* strain Bl21 Star^TM^ (DE3) and purified as previously described^23^. Pure samples were dialysed against 20 mM PIPES, pH 7.0 and quantified using the theoretical extinction coefficients ε_279_=27.8 mM^−1 ^cm^−1^ and 28.8 mM^−1 ^cm^−1^ for *Ca*FADS and *Spn*FADS, respectively. The purity of each protein was tested by 15% SDS-PAGE.

### Chemicals

The Prestwick Chemical Library^®^, containing 1240 molecules approved by the Food and Drugs Administration (FDA) and European Medicines Agency (EMA), was selected for the HTS. Compounds were dissolved in 100% DMSO at 10 mM. All the HTS hits were subsequently acquired from Sigma Aldrich, Prestwick or Carbosynth and dissolved in 100% DMSO to prepare stock solutions at 50 and 10 mM. The purity of all compounds was >95%, as determined by High performance liquid chromatography (HPLC), thin layer chromatography (TLC), NMR, IR or basic titration.

### Activity-based high-throughput screening for CaFADS

An activity-based HTS was performed on the 1240 compounds of the Prestwick Chemical Library^®^. The assays consisted in recording the time dependent decrease in the fluorescence of the isoalloxazine ring, produced upon transformation of RF and FMN into FAD, as a consequence of the fluorescence quenching in this later flavin^27^. When either the RFK or the FMNAT activities were inhibited, less FAD was produced and, consequently, the fluorescence decrease registered in a specific time interval was less pronounced. Measurements were carried out using a multimode microplate reader, Synergy™ HT Biotek, with BRAND 96-well plates pure Grade™. To optimise the assay conditions, a previous study was performed using constant concentrations of RF, ATP and *Ca*FADS (∼5, ∼50 and ∼0.4 µM, respectively) and variable concentrations of MgCl_2_ (0.2–10 mM) and DMSO (0–12.5% v/v). Optimum conditions were 2.5% DMSO, 10 mM MgCl_2_ and sensitivity 70.

HTS reaction mixtures contained 5 µM RF, 0.4 µM *Ca*FADS, 10 mM MgCl_2_, in PIPES 20 mM, pH 7.0, 2.5% DMSO, and the corresponding compound of the chemical library at a final concentration of 250 µM. Reactions were initiated through addition of 50 µM ATP, being the final reaction volume 100 µl. Controls, which contained the reaction mixture but not any chemical from the library, were added both to the first and last columns of the plate. Flavin fluorescence in each well was registered at 25 °C, every 50 s during 15 min. Excitation and emission wavelengths were 440 and 530 nm, respectively. The slope of the resulting line, recorded between 0 and 6 min, was calculated for every compound, and also for the controls, as well as the fluorescence change per time unit (Δ*F*/Δ*t*). The compounds that decreased the reaction rate below the average reaction rate of the controls minus the standard deviation could be preselected as potential inhibitors, but we reduced further the cut-off by selecting only those compounds inhibiting more than 50% of the controls activity as HTS hits.

### Identification of the activity inhibited by each of the HTS hits

The decreasing of the reaction rate by the presence of the HTS hits might be consequence of the compounds inhibiting the RFK activity, the FMNAT one, or both of them; also, it could be a false positive due to the properties of pan assay interference compounds (PAINS). To clarify this point, we first checked that there were no PAINS among the HTS hits using the FAF-Drug4 web server^31^. Then, the RFK and FMNAT reactions were individually assayed in the presence of the HTS hits at 25 °C. Reaction mixtures contained 50 µM ATP, 5 µM RF, 0.4 µM *Ca*FADS in 20 mM PIPES, pH 7.0, 0.8 mM MgCl_2_, when assaying the RFK activity, and 50 µM ATP, 10 µM FMN, 0.4 µM *Ca*FADS in 20 mM PIPES, pH 7.0, 10 mM MgCl_2_ when measuring the FMNAT reaction. Each HTS hit was tested again at 250 µM for each of the two enzymatic reactions. Finally, reactions were stopped by boiling the samples at 100 °C for 5 min, and the precipitated protein was eliminated through centrifugation. The transformation of RF into FMN or FAD was evaluated through flavins separation by HPLC, as previously described^27^. Those HTS hits decreasing the FMNAT activity by more than 95% of the controls, without significantly affecting the RFK activity (rates over 75% those of the controls) were selected as FMNAT hits for further assessment. When assaying the HTS hits against the FMNAT activity of *Spn*FADS, similar conditions were used but samples contained 3 mM sodium dithionite to maintain the flavin in its reduced state^23^. Data were processed as previously reported^27^. All the experiments were performed in triplicate.

### Determination of the potency of FMNAT hits

To determine the IC_50_ values of the FMNAT hits, the FMNAT activity was assayed at different concentrations of each inhibitor (0–100 µM range) and 25 °C. Experiments were performed and analysed through HPLC as described above. Positive controls (without any hit compound) were included in every reaction set. DMSO concentration was kept at 2.5% in all samples. All the experiments were performed in triplicate.

### Determination of the inhibition mechanism of CaFADS by FMNAT hits 24, 27 and 31

The inhibition mechanism was further studied for the three FMNAT hits that showed the lowest IC_50_ and minimal residual FMNAT activities, namely, 24, 27 and 31. Reaction mixtures containing 0–100 µM of each compound, 1–20 µM FMN and 400 µM ATP were used when analysing the inhibitory effect of the compound regarding the FMN substrate, while 5–400 µM ATP and 15 µM FMN when analysing the effect of the inhibitor regarding the ATP substrate. All the experiments were carried out in 20 mM PIPES, 10 mM MgCl_2_, pH 7.0, 2.5% DMSO at 25 °C, being the final reaction volume 500 µl. The reactions were initiated by addition of *Ca*FADS at a final concentration of ∼40 nM, followed by 1 min incubation. The flavin composition of the supernatant was analysed as previously described^27^. All the experiments were performed in triplicate. The effect of the inhibitors on *K*_m_ and *V*_max_ was determined by fitting the data sets to the Michaelis–Menten model. Additionally, data were globally fit to Lineweaver–Burk equations for competitive, uncompetitive, non-competitive or mixed inhibition, yielding *K_i_*, as well as *K_i_′* when applying, for each compound (Equations (1–4), respectively).

(1) $$\frac{1}{V_{0}}=\frac{\left(1+\frac{\left[I\right]}{K_{i}}\right)\cdot K_{m}}{V_{max}}\cdot \frac{1}{\left[S\right]}+\frac{1}{V_{max}}$$

(2) $$\frac{1}{V_{0}}=\frac{\left(1+\frac{\left[I\right]}{{K'}_{i}}\right)}{V_{max}}+\frac{K_{m}}{V_{max}}\cdot \frac{1}{\left[S\right]}$$

(3) $$\frac{1}{V_{0}}=\frac{\left(1+\frac{\left[I\right]}{K_{i}}\right)}{V_{max}}+\frac{{\left(1+\frac{\left[I\right]}{K_{i}}\right)\cdot K}_{m}}{V_{max}}\cdot \frac{1}{\left[S\right]}$$

(4) $$\frac{1}{V_{0}}=\frac{\left(1+\frac{\left[I\right]}{K_{i}}\right)\cdot K_{m}}{V_{max}}\cdot \frac{1}{\left[S\right]}+\frac{\left(1+\frac{\left[I\right]}{{K'}_{i}}\right)}{V_{max}}$$

### Thermodynamic characterisation of binding of hits 24, 27 and 31 through isothermal titration calorimetry (ITC)

ITC experiments were performed to characterise the protein’s affinity for the selected compounds, as also the thermodynamic parameters that drive the interaction. Experiments were carried out in an AutoITC200 (*MicroCal*) thermostated at 25 °C. In these experiments, ∼400 µM of each compound were used to titrate ∼25 µM *Ca*FADS contained in a 200 µL cell. However, when saturation of the protein was not reached, higher concentrations of compounds were employed. The titrations were performed by stepwise injections of the titrating compound. Up to 19 injections of 2 µl were added to the cell sample and mixed using a 1000 rpm stirrer syringe. The compounds and the protein were dissolved in 20 mM PIPES, pH 7.0, 10 mM MgCl_2_ and degassed prior to titration. DMSO was added to the protein and ligand samples, until reach a final concentration of 3%. The association constant (*K*_a_), the enthalpy variation (Δ*H*) and the binding stoichiometry (N), were obtained through non-lineal regression of the data to a model for one or two independent binding sites, implemented in Origin 7.0 (*OriginLab*) as previously described^27^^,^^29^. The entropic contribution (−TΔS), the Gibbs free energy (Δ*G*) and the dissociation constant (*K*_d_) were obtained through essential thermodynamic equations.

### Docking of hits 24, 27 and 31 to the FMNAT module of CaFADS

The AutoDock4.2 software^32–34^ and the coordinates of a monomer from *Ca*FADS (PDB 2X0K)^24^ were used to obtain the interaction models with 24, 27 and 31. The space sampling was defined using a grid box of 90 points in each dimension, and placing the H57 NE atom of the FMNAT module as the grid box centre. The grid size was 0.375 Å. The search was performed using the lamarkian genetic algorithm, with a starting population of 150 individuals, using 25,000,000 energy evaluations and 27,000 generations. The 24, 27 and 31 initial structures for the docking protocol were optimised using the functional B3LYP with the basis set 6–31 G (d,p) and the gaussian09 software^35^. The structural poses with the lowest docking score were selected and analysed.

### Determination of the antibacterial activity of the HTS hits

The antimicrobial activity of the HTS hits was tested by the colorimetric method of the resazurin microplate assay^36^ according to broth microdilution method guidelines (CLSI; Clinical and Laboratory Standards Institute). Serial 2-fold dilutions of the HTS hits were performed in BHI medium, in 96-well microtiter plates, with a final volume of 100 µl per well. Subsequently, liquid cultures of *C. ammoniagenes* ATCC 6872 in logarithmic phase were adjusted to 10^6^ CFU/ml in BHI broth, and 100 µl of this suspension were added to each well, making a final inoculum of 5 × 10^5^ CFU/ml. Plates were incubated 16 h at 37 °C. 30 µl of 0.1 mg/ml resazurin solution were then added to each well, and results were observed after 4 h of incubation at 37 °C. Resazurin (blue) is an indicator of bacterial growth, since metabolic activity of bacteria reduces it to resorufin (pink). The minimum inhibitory concentration (MIC) is the lowest concentration of compound that does not change the resazurin colour from blue to pink.

Similarly, the HTS hits were also assayed at 37 °C against *M. tuberculosis* ATCC 27,294 and *S. pneumoniae* ATCC 49619 cells. In these experiments the initial cell concentration was also 5 × 10^5^ CFU/ml, and plates were incubated for 10 h (*S. pneumoniae*), and 6 days (*M. tuberculosis*) before addition of resazurin. Results were observed after incubation with resazurin 4 h and two days for *S. pneumoniae* and *M. tuberculosis*, respectively. In these experiments, culture media were; Middlebrook 7H9 (Difco) supplemented with 10% ADC (0.2% dextrose, 0.5% V fraction BSA and 0.0003% bovine catalase) (BD Difco) and with 0.5% glycerol (Scharlau) for *M. tuberculosis* growth, and BHI supplemented with 4% FBS (Gibco) for *S. pneumoniae* growth.

### Statistics

Results are expressed as the mean ± the standard deviation (SD) or as the mean ± the standard error (SE) of the regression. When indicated, one-way analysis of variance (ANOVA) was performed to determine statistical significance.

## Results

### Identification of potential inhibitors of the CaFADS activities through HTS

**Figure 1.**
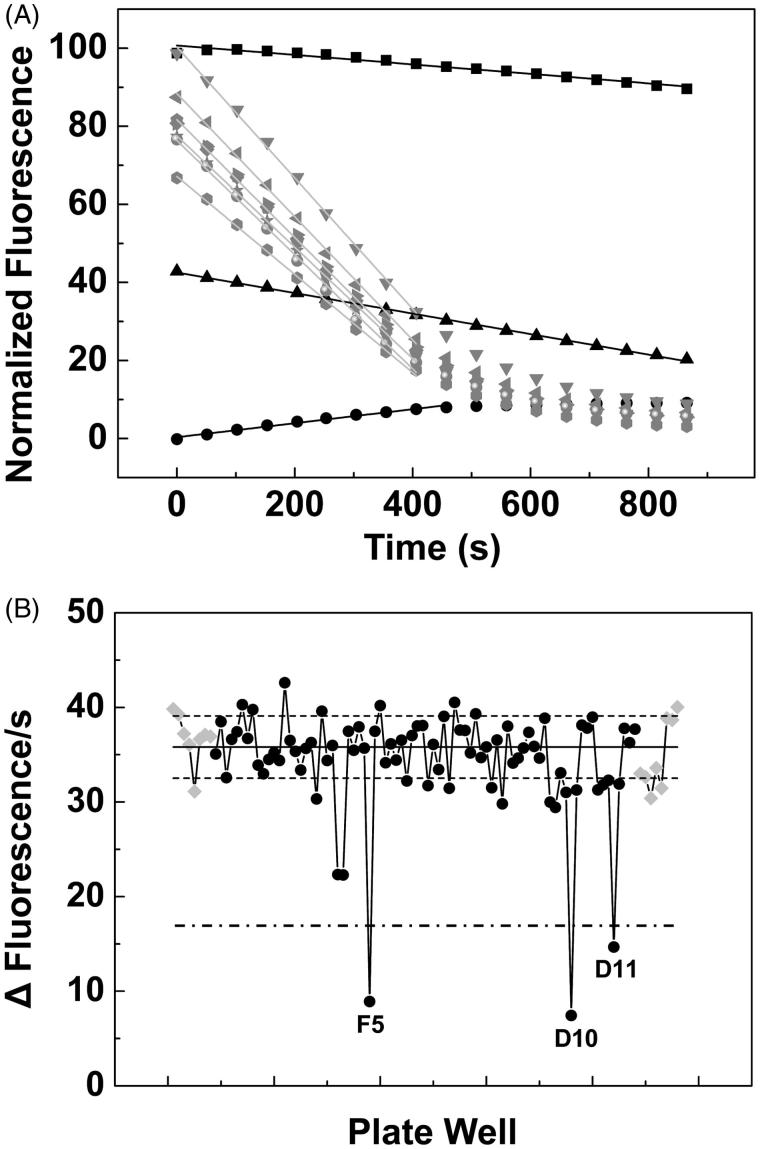
Activity-based high throughput screening (HTS) for the discovery of inhibitors of the RFK and/or the FMNAT activities of *Ca*FADS. (A) Example of the flavin fluorescence evolution over time for three of the identified hits and for control assays. Reaction mixtures were incubated at 25 °C and contained 5 µM RF, 50 µM ATP, 0.4 µM *Ca*FADS, 10 mM MgCl_2_, in PIPES 20 mM, pH 7.0, 2.5% DMSO. The black symbols and lines correspond to kinetic traces at wells containing library compounds at 250 µM, while grey ones correspond to control wells. (B) Initial velocities (Δfluorescence/s) for the reactions in each of the wells of a HTS plate. Data from wells containing chemical library compounds are in black while controls are in grey. The solid line represents the average velocity obtained for the positive controls of the reaction and the dotted lines are the average velocity plus and minus the standard deviation. The letters and numbers indicate the position of the well in the plate (row and a column respectively) for each specific selected measurement. A bold dashed line indicates 50% the rates of controls.

To identify potential inhibitors of the *Ca*FADS activities we designed the activity-based HTS assay described in the methods section, which allowed to determine rates for the transformation of RF into FAD (via the FMN intermediate) in each one of the plate wells. Wells containing chemicals of the library and decreasing reaction rates relative to the controls (absence of chemicals) were preselected as containing compounds that are potential inhibitors of at least one of the *Ca*FADS activities. Thus, among the 1240 compounds of the chemical library, 140 (13.5%) reduced the *Ca*FADS activity levels below the mean of the positive controls minus twice its standard deviation, and of them, 37 (3.6%) reduced the positive controls average rate for FAD formation in a factor higher than 0.5 (Figure 1). Those 37 compounds were selected as the HTS hits (Supplementary Chart SD1).

### Effect of the HTS hits on the RFK and FMNAT activities of CaFADS

**Figure 2.**
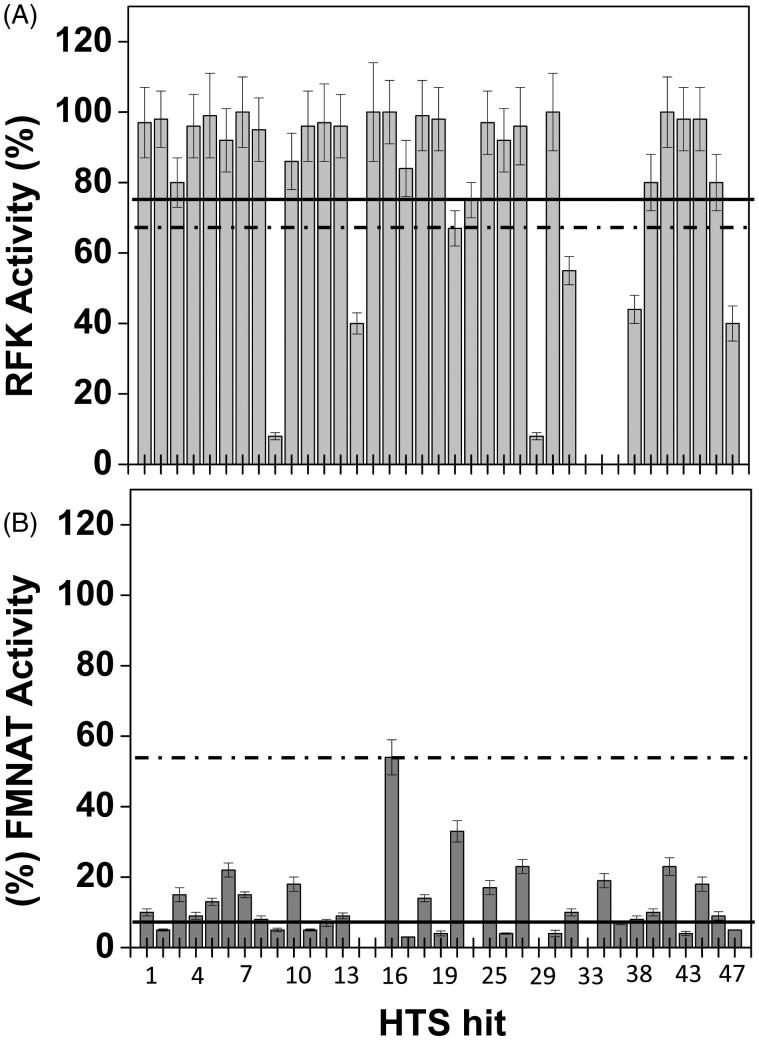
Effect of the HTS hits on the RFK and FMNAT activities of *Ca*FADS. Residual (A) RFK and (B) FMNAT activities when assayed in the presence of 250 μM of the 37 HTS hits. In (A), the columns below the dashed line present statistical significant inhibition by the corresponding hit (*p* < 0.002, 67% remaining activity) related to the control *Ca*FADS RFK activity. In (B), all hits produce statistical significant inhibition (*p* < 0.0001, dashed line) when compared with the controls of the *Ca*FADS FMNAT activity. Solid lines indicate 75 and 5% of the control RFK and FMNAT activities, respectively. The HTS hits displaying <5% and >75% of the control FMNAT and RFK activities, respectively, were selected for further study. Experiments carried out in 20 mM PIPES, pH 7.0, 2.5% DMSO at 25 °C, with 7.5 μM RF, 350 μM ATP, 0.8 mM MgCl_2_ (for the RFK activity) or 15 μM FMN, 350 μM ATP, 10 mM MgCl_2_ (for the FMNAT activity) (*n* = 3; mean ± SD).

**Chart 1.**
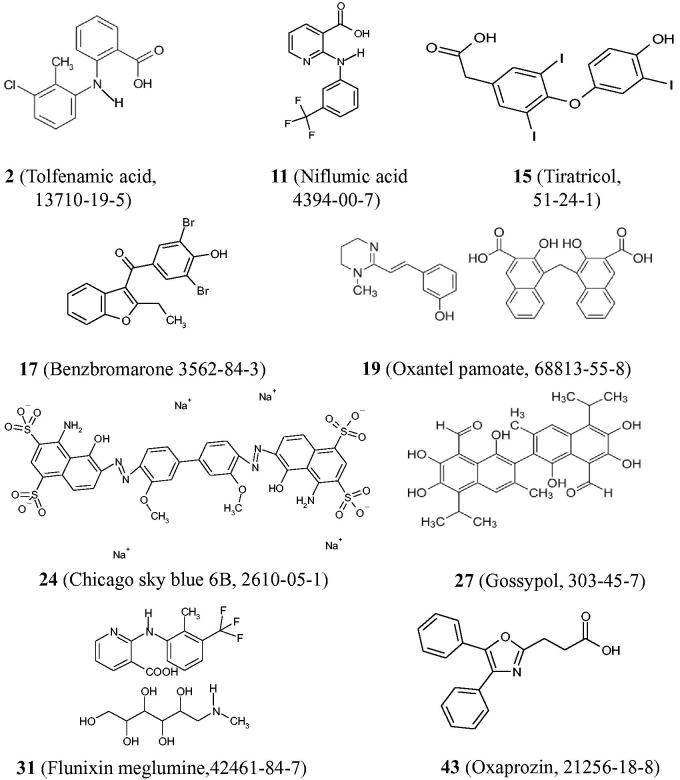
Chemical structures of compounds selected as FMNAT hits for *Ca*FADS.

We then move to identify which one of the activities (RFK or FMNAT), and in which extension, was affected by each one of these 37 HTS hits. With this aim, we assayed the effect of the HTS hits both on the *Ca*FADS RFK and FMNAT activities. Figure 2 and Table 1 summarise the results. Comparison of Figure 2(A,B) shows that, in general and under the assay conditions, the 37 HTS hits produced a stronger deleterious effect on the FMNAT activity (all decreased the activity of the controls in more than 50%) than on the RFK one. The FMNAT module of *Ca*FADS does not have sequence or structural homology with the mammalian protein but the RFK module belongs to the eukaryotic RFKs family. Therefore, we decided to continue the study with the HTS hits that inhibit the FMNAT activity, since they are more likely to be specific to the bacterial proteins. Thus, we choose the HTS hits that decreased the FMNAT activity below 5% of that of the controls, but maintained over 75% the RFK activity (Figure 2, Table 1). Thus, among (2)\(11), tiratricol (15), benzbromarone (17), oxantel pamoate (19), Chicago sky blue 6B (24), gossypol (27), flunixin meglumine (31) and oxaprozin (43) (Chart 1) were selected as FMNAT hits. It is worth noticing that although some of these compounds are apparently structurally related with other of the HTS hits, slight differences in functional groups and geometries induce different enzymatic responses in the FMNAT or RFK activities. This is a fact of particular interest when developing specific inhibitors.

**Table 1.** RFK and FMN residual activities of *Ca*FADS in the presence of the HTS hits.

		% Residual RFK activity			
		≤ 5	5–50	50–75	≥75
% Residual FMNAT activity					
≤5		9, 29, 33, 37	14,47		*2, 11, 15, 17, 19, 24, 27, 31, 43*
5–50		35	38	22,32	1, 3, 4, 5, 6, 7, 8, 10, 12, 13, 16, 18, 25, 28, 39, 40, 44, 46
≥50		–	–	–	–

Values measured at 25 °C, in 20 mM PIPES, pH 7.0, 2.5% DMSO and 0.8 or 10 mM MgCl_2_ when assaying the RFK or the FMNAT activities, respectively. The final concentration of each HTS hit was 250 μM and saturating concentrations of all the substrates were used. The compounds highlighted in italics completely inhibited the FMNAT activity with minor effects on the RFK one, and were selected as FMNAT hits.

**Figure 3.**
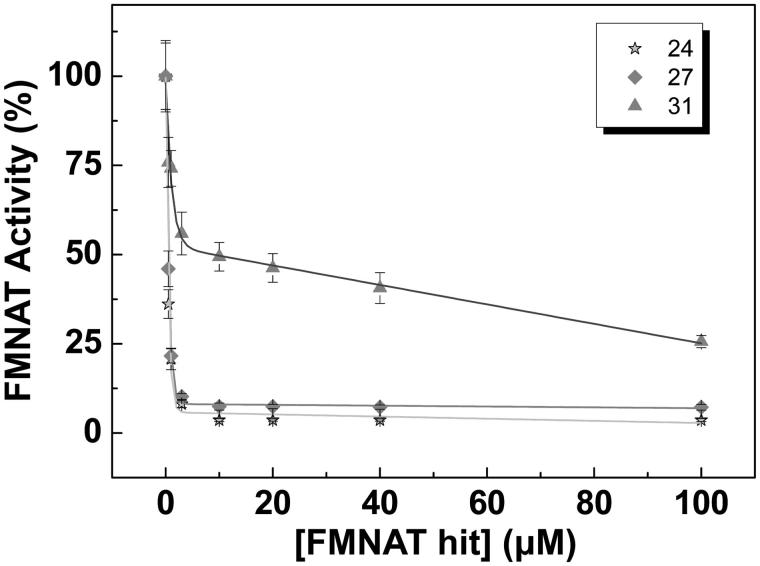
Dose–response curves for the FMNAT activity of *Ca*FADS in the presence of representative hits. Values derived from these representations are included in Table 2. Experiments performed at 25 °C in 20 mM PIPES, pH 7.0, 10 mM MgCl_2_, 2.5% DMSO, with 15 μM FMN and 350 μM ATP (*n* = 3, mean ± SD).

To rate the power of these 9 FMNAT hits as inhibitors of the *Ca*FADS FMNAT activity, their half maximal inhibitory concentrations (IC_50_) and the remaining activity at 50 µM of each compound were determined (Figure 3, Table 2). The 9 compounds yielded IC_50_ values in the micromolar range, reducing by more than half the activity of the controls. Considering both their IC_50_ value and residual activity, the most potent inhibitors were 24 (IC_50_ = 0.4 ± 0.1 µM), 27 (IC_50_ = 0.5 ± 0.1 µM) and 31 (IC_50_ = 6.6 ± 0.6 µM). These three compounds produce residual activities below 25% of the controls. Compound 43 also showed high inhibitory potency, but due to its low solubility in the working buffer it was discarded.

**Table 2.** Effect of the FMNAT hits on the FMNAT activity of *Ca*FADS.

FMNAT hit	Residual activity^a^ (%)	IC_50_^b^ (μM)
2	41.1 ± 5.2	8.9 ± 1.0
11	34.6 ± 4.9	9.0 ± 1.3
15	45.3 ± 4.2	40.7 ± 3.9
17	33.5 ± 10.1	12.8 ± 3.4
19	43.3 ± 3.9	20.8 ± 2.6
24	3.6 ± 0.2	0.4 ± 0.1
27	6.9 ± 0.8	0.5 ± 0.1
31	24.5 ± 1.9	6.6 ± 0.6
43^c^	20.3 ± 5.2	1.0 ± 0.5

Experiments carried out at 25 °C, in 20 mM PIPES, pH 7.0, 10 mM MgCl_2_ at saturating FMN and ATP. All samples contained 2.5% DMSO (*n* = 3, mean ± SD).

a Remaining activity in the presence of 50 μM of each compound. All data show statistical significance differences when compared with activity in the compound absence (***p < 0.0001).

b Compounds assayed in the 0–100 μM concentration range.

c This compound shows very low water solubility, so it was discarded to continue the study even though its good properties.

### The inhibition mechanisms of 24, 27 and 31

**Figure 4.**
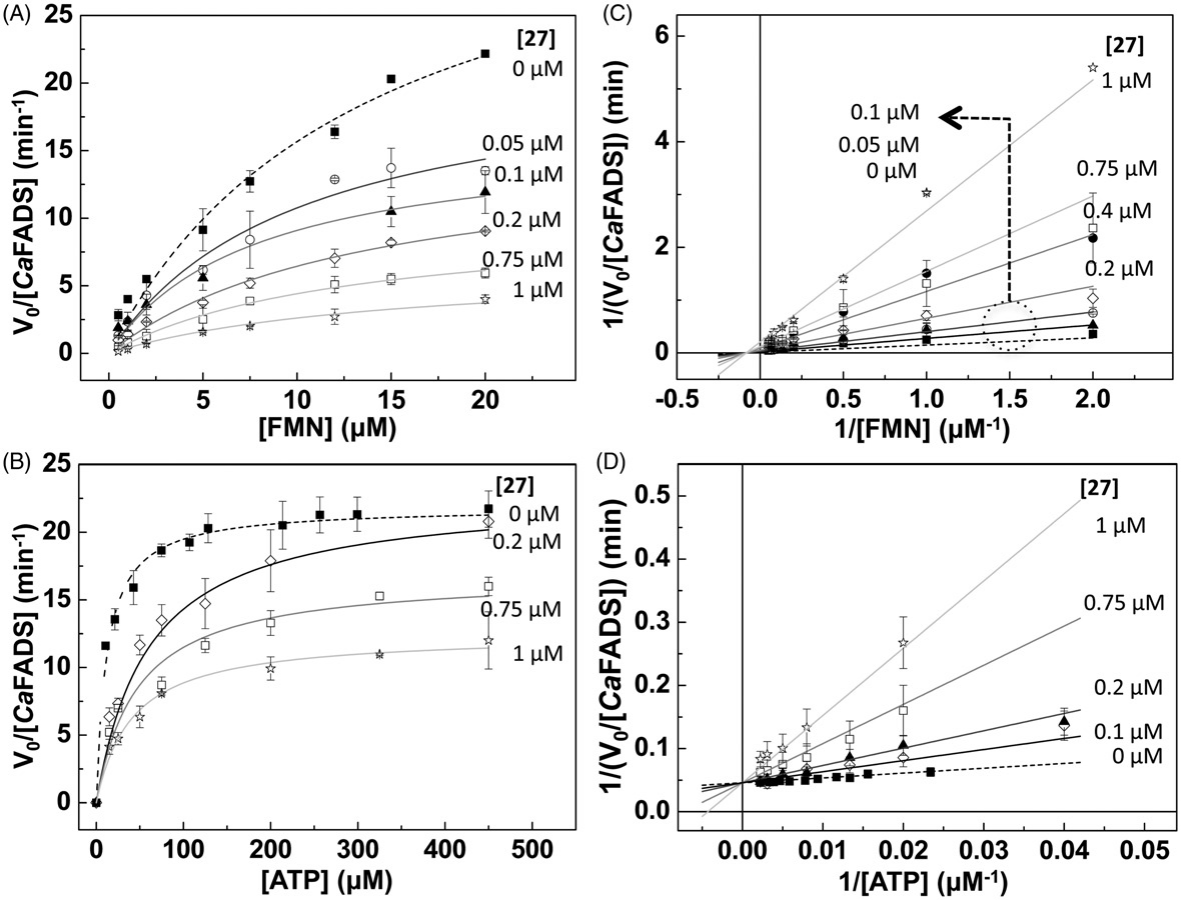
Hit 27 as an inhibitor of the FMNAT activity of *Ca*FADS. Michaelis–Menten plots at different concentrations of 27 and saturation (A) of ATP and (B) of FMN. Lineaweaver–Burk representations with global fit to (C) non-competitive inhibition at saturating ATP and (D) competitive inhibition at saturating FMN. Reaction rates obtained in 20 mM PIPES, pH 7.0, 10 mM MgCl_2_, at 25 °C, with 15 μM FMN and 10–450 μM ATP (FMN saturating) or with 350 μM ATP and 0.5–20 μM FMN (ATP saturating). All samples contained 2.5% DMSO (*n* = 3, mean ± SD).

To determine the inhibition mechanism of the hits 24, 27 and 31, we measured the FMNAT activity of *Ca*FADS in the presence of increasing concentrations of each compound. Considering that this is a bi-substrate activity, for each compound we carried out two set of experiments at; (i) saturating ATP and different FMN concentrations, and (ii) saturating FMN and increasing ATP concentrations. Then we fit our experimental data to the Michaelis–Menten model, obtaining *K*_m_ and *k*_cat_ values. The analysis of the evolution of these constants on the hits concentrations, together with the corresponding Lineawever–Burk plots [representation of data as double inverses and fit to Equations (1–4)] (Figure 4, Supplementary Figures SD2 and SD3, Supplementary Table SP1) allowed identifying the inhibitory mechanisms of the 24, 27 and 31 hits, as well as the corresponding inhibition constants (*K_i_* or, *K_i_* and *K_i_′*) (Table 3). The experiments carried out at saturating ATP (varying the FMN concentrations), revealed that the three compounds are strong non-competitive inhibitors of the *Ca*FADS FMNAT activity regarding the FMN substrate (*K_i_*values around 0.08 µM, Table 3). Nevertheless, when using a constant and saturating FMN concentration but varying the ATP concentration, their inhibition mechanisms differ among them. 24 is a strong ATP uncompetitive inhibitor (*K_i_*= 0.08 ± 0.03 µM), therefore, it is able to bind the *Ca*FADS-ATP complex and reduce the amount of enzyme that is available to react. 27 is a strong competitive inhibitor regarding the ATP substrate (*K_i_* = 0.06 ± 0.01 µM), while 31 is a considerably poorer mixed inhibitor. Thus, 31 is able to bind to both the free enzyme and the *Ca*FADS-ATP complex, although binding constants indicate that binding to free *Ca*FADS is preferred (*K_i_* 3.5 ± 1.0 µM versus *K_i_′* 18.4 ± 4.0 µM, Table 3).

**Table 3.** Inhibition constants and mechanisms of the best FMNAT hits relative to the FMNAT activity of *Ca*FADS.

	Saturating ATP		Saturating FMN		
	*K_i_* (μM)	Inhibition mechanism	*K_i_* (μM)	*K^’^_i_* (μM)	Inhibition mechanism
24	0.07 ± 0.01	Non-competitive	0.08 ± 0.03	–	Uncompetitive
27	0.08 ± 0.01	Non-competitive	0.06 ± 0.01	–	Competitive
31	0.09 ± 0.03	Non-competitive	3.5 ± 1.0	18.4 ± 4.0	Mixed

Experimental data recorded at 25 °C in 20 mM PIPES, pH 7.0, 10 mM MgCl_2_ and 2.5% DMSO. Data obtained by globally fitting the experimental data to the corresponding Lineweawer–Burk inhibition model.

### Binding of 24, 27 and 31 to CaFADS

**Figure 5.**
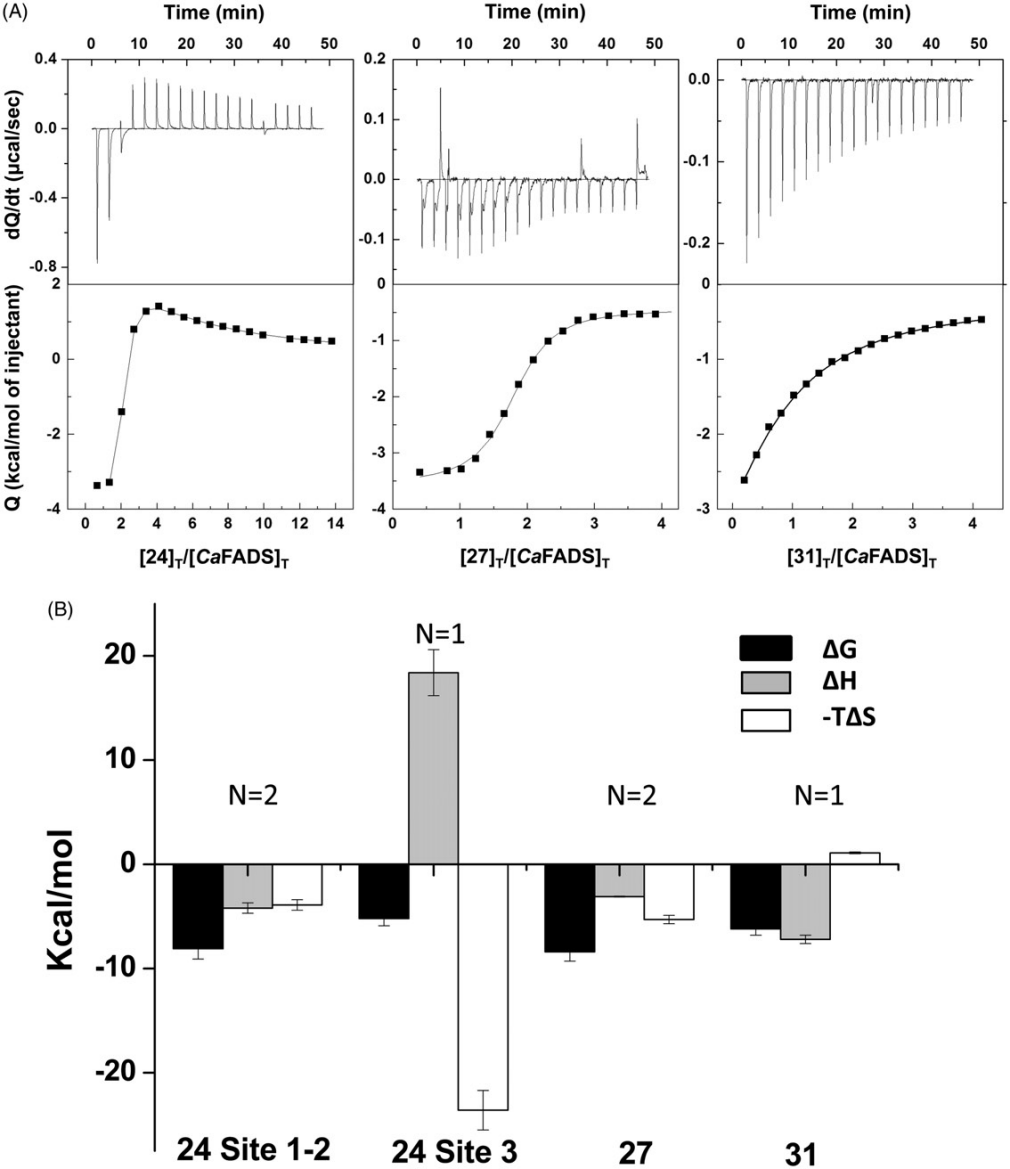
Thermodynamic analysis of the binding of the selected FMNAT hits to *Ca*FADS. (A) Calorimetric titrations for the 24, 27 and 31 compounds. The upper panels show the thermograms for the interaction and the lower panels show the corresponding binding isotherms with integrated heats. (B) Thermodynamic dissections of the interaction of *Ca*FADS with each of the selected compounds. The binding Gibbs energy (Δ*G*), enthalpy (Δ*H*) and entropy (−TΔ*S*) are represented in black, grey and white bars, respectively. Experiments were carried out at 25 °C, in 20 mM PIPES, pH 7.0, 10 mM MgCl_2_ and 3% DMSO.

The interaction of *Ca*FADS with compounds 24, 27 and 31 was characterised using ITC. This is a very powerful technique because first the shape of the thermograms informs us about the number of binding sites related to the thermodynamic nature of binding. Then, fitting of experimental data to the equations describing binding models allow determining thermodynamic binding parameters, as well as binding stoichiometry at each of the different binding sites. Thus, analysis of our ITC titrations indicates that the three compounds bind the enzyme at, at least, one binding site (Figure 5(A), Table 4). Thus, we identified in *Ca*FADS a unique binding site of moderate-low affinity for 31 (*N* ≈ 1, *K*_d_ = 30.9 ± 2.8 µM) and two binding sites of high and similar affinity for 27 (*N* ≈ 2, *K*_d,av_ = 0.7 ± 0.07 µM, this *K*_d,av_ value is an average value, since the similarity between the two binding sites prevents them to be distinguished). These 27 and 31 binding sites are expected to be located at the enzyme FMNAT domain, since that is the inhibited activity. The interaction between the hit 24 and the enzyme resulted more complicated, because our ITC data indicate that compound 24 binds to the protein at three sites. Two of them show similar and strong affinity (*K*_d,av_ = 1.1 ± 0.1 µM) and therefore, we suggest that they might be located at the FMNAT module, since that is the activity mainly inhibited by compound 24. The third site for 24 binding has lower affinity (*K*_d_ = 161 ± 20 µM). Since this compound also mildly inhibits the RFK activity, we presume this third binding site may be at the RFK module. All bindings here characterised (with the only exception of the low affinity binding site for 24) are favoured by the enthalpic contribution (Figure 5(B), Table 4). This suggests a net gain of H-bonding and ion-pair interactions and indicates specific binding interactions. Regarding the entropic contribution to the binding free energy, it is small and favourable for 24 and 27, and only slightly unfavourable for 31 (Figure 5(B), Table 4). However, it drives the binding of 24 to the RFK module (site 3 Figure 5(B)), revealing that it might be non-specific and that could occur due to the compound hydrophobicity and rigidity^37^.

**Table 4.** Thermodynamic parameters for the interaction of *Ca*FADS with the hits **24**, **27** and **31**.

Hit	N	*K*_d_ (μM)	Δ*G* (kcal/mol)	Δ*H* (kcal/mol)	−*T*Δ*S* (kcal/mol)	Docking Δ*G*^b^ (kcal/mol)
24						
Site 1–2	≈ 2	1.1 ± 0.1^a^	−8.1 ± 1.0^a^	−4.2 ± 0.5^a^	−3.9 ± 0.5^a^	−8.2 ± 0.5/-4.2 ± 0.4
Site 3	≈ 1	161 ± 20	−5.2 ± 0.7	18.4 ± 2.2	−23.6 ± 1.9	n.d.^c^
27	≈ 2	0.7 ± 0.07^a^	−8.4 ± 0.9^a^	−3.1 ± 0.1^a^	−5.3 ± 0.4^a^	−11.9 ± 0.1/−9.1 ± 0.1
31	≈ 1	30.9 ± 2.8	−6.2 ± 0.6	−7.2 ± 0.4	1.1 ± 0.1	−6.59 ± 0.1

ITC experiments performed at 25 °C, in PIPES 20 mM, pH 7.0, 10 mM MgCl_2_, 3% DMSO.

a These parameters correspond to average values for the binding of two hit molecules.

b Docking score for the best pose, with standard deviation for the best five poses.

c Not calculated.

**Figure 6.**
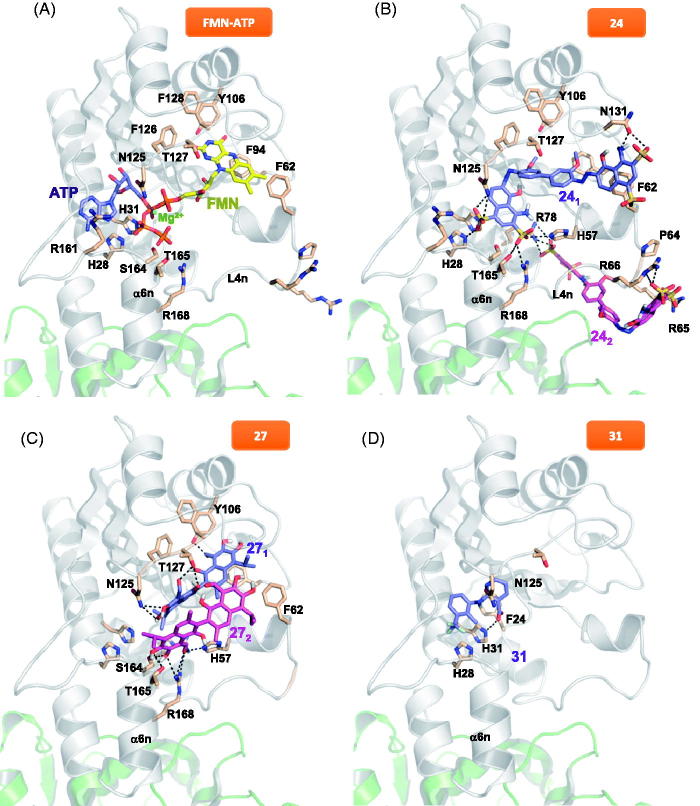
Docking models for the binding conformations of the selected FMNAT hits to the *Ca*FADS FMNAT module. (A) Model of the theoretical placement of substrates. ATP in violet, Mg^2+^ as a green dot and FMN in yellow. Data from (24). Best docking pose of (B) FADS:24_1_ and FADS:24_1_:24_2_ models (24_1_ in violet, 24_2_ in pink), (C) FADS:27_1_ and FADS:27_1_:27_2_ models (27_1_ in violet, 27_2_ in pink), and (D) FADS:31 (31 in violet, docking corresponds only to the flunixin bioactive part of 31, meglumine is the excipient). Side-chains of key residues are shown as CPK sticks with carbons in wheat. H-bonds are indicated as dashed lines. The protein is shown as a cartoon, having the FMNAT and RFK modules coloured in grey and green, respectively. Docking was performed using Autodock4.2.

To investigate how the FMNAT module of *Ca*FADS accommodates these compounds, we performed a computational protein-ligand docking (Figure 6 and Supplementary Figure SD4). In the highest-scoring docking mode, as well as for the best five poses (purple molecule in Figure 6(B), Supplementary Figure SD4, respectively), 24_1_ (1 indicates the first molecule of 24 docked) is situated in the substrates binding pockets (Figure 6A)^24^. One of the 24_1_ moieties binds through one of its sulphates to S164 and H31. 24_1_ is in addition H-bonded to the N125 catalytic base^17^, as well as to Y106, T127 and N131 at the loop that forms the upper flavin ring binding site (Figure 6(B)). Since our ITC data suggest two binding sites for compound 24 at the FMNAT module, we carried out an additional docking analysis to identify the second site, 24_2_ (pink molecule in Figure 6(B)), using as receptor our highest-scoring FADS:24_1_ model. Considering the binding energies (Table 4) this site is expected to be less populated, but given that 24 is an ATP uncompetitive inhibitor, the docking binding energy could improve if we consider the ATP substrate presence instead of the 24_1_ molecule. Additionally, it is worth noting that in the presence of substrates or products, the 24 binding conformations might differ from the ones presented here. In the most favourable docking poses, the first molecule of 27 bound to the protein (27_1_) is H-bonded by T127 and the N125 catalytic base at the binding site of the ATP phosphates^17^ (purple molecule in Figure 6(C) and SD4), in agreement with 27 being an ATP competitive inhibitor. Because two binding sites were again predicted by ITC at the FMNAT site, we carried out a second docking using as receptor the highest-scoring FADS:27_1_ model. 27_2_ is stabilised by H-bonds with the N-terminal of the α6n helix, T165 and R168, at the ATP binding site entrance (pink molecule in Figure 6(C)). We observed direct interaction between the two 27 molecules, and 27_2_ somehow resembling the 24_1_ binding (purple molecule in Figure 6(B)). For 31, we only carried out the flunixin docking, given that this compound is the bioactive agent of 31 and meglumine is the excipient. The best poses for 31 binding are suggested at the ATP binding site (Figure 6(D) and SD4), stabilised by a H-bond with H31. This binding is coherent with the ATP mixed inhibition mechanism of 31. In all cases, the H-bond interactions explain the favourable enthalpic binding contributions revealed by ITC, while the favourable entropic contributions can be attributed to the expelling of structural water molecules from the binding cavities, particularly at the substrates binding sites.

### Effect of the HTS hits on the RFK and FMNAT activities of SpnFADS

To determine whether the 37 HTS hits were specific for *Ca*FADS or might have effect on other similar bacterial FADS family members, we tested their effects on the RFK and FMNAT activities of the also bimodular and bifunctional *Spn*FADS. Eleven of the 37 HTS hits inhibited either the RFK or the FMNAT activities of *Spn*FADS (Table 5). However, most compounds exhibited IC_50_ in the high micromolar range (>65 µM). Only fluvastatin sodium salt (37) for the FMNAT activity, and thonzonium bromide (9) and 27 for the RFK one showed IC_50_ lower than 10 µM. 37 (IC_50_ = 7 ± 1 µM) and 9 (IC_50_ = 6 ± 1 µM) inhibit completely the corresponding activity, but the residual RFK activity with 27 was too high to be considered as a good inhibitor.

**Table 5.** Effect of selected HTS hits on the RFK and FMNAT activities of *Spn*FADS.

	RFK activity		FMNAT activity	
HTS hit	Res. activity^a^ (%)	IC_50_^b^ (μM)	Res. activity^a^ (%)	IC_50_^b^ (μM)
1	100 ± 15	–	0 ± 0***	69 ± 5
2	78 ± 10	>100	100 ± 12	–
7	92 ± 10	>100	0 ± 0***	73 ± 7
9	0 ± 0***	6 ± 1	7 ± 1***	78 ± 6
10	100 ± 12	–	0 ± 0***	68 ± 7
14	100 ± 10	–	63 ± 7***	>100
24	84 ± 9	>100	0 ± 0***	70 ± 6
25	81 ± 8	>100	0 ± 0***	70 ± 6
27	25 ± 4 ***	6 ± 1	0 ± 0 ***	51 ± 6
29	71 ± 8*	>100	0 ± 0 ***	64 ± 5
33	26 ± 3 ***	33 ± 4	93 ± 11	>100
37	96 ± 10	>100	0 ± 0 ***	7 ± 1
38	40 ± 5 ***	88 ± 6	68 ± 7 **	>100
43	32 ± 3 ***	14 ± 2	100 ± 15	–

All the experiments were carried out at 25 °C, in 20 mM PIPES pH 7.0, 10 mM MgCl_2_ at saturating concentrations of FMN and ATP and in the presence of 2.5% DMSO. (*n* = 3, mean ± SD).

a Remaining activity in the presence of 100 μM of each compound. Data showing statistical significance differences when compared with activity in the absence of compound (***p < 0.0001; **0.0021 > *p* > 0.0001; *0.033 > *p* > 0.0021).

b Compounds assayed in the 0–100 μM concentration range.

### Effect of selected HTS hits on different bacterial cells

**Figure 7.**
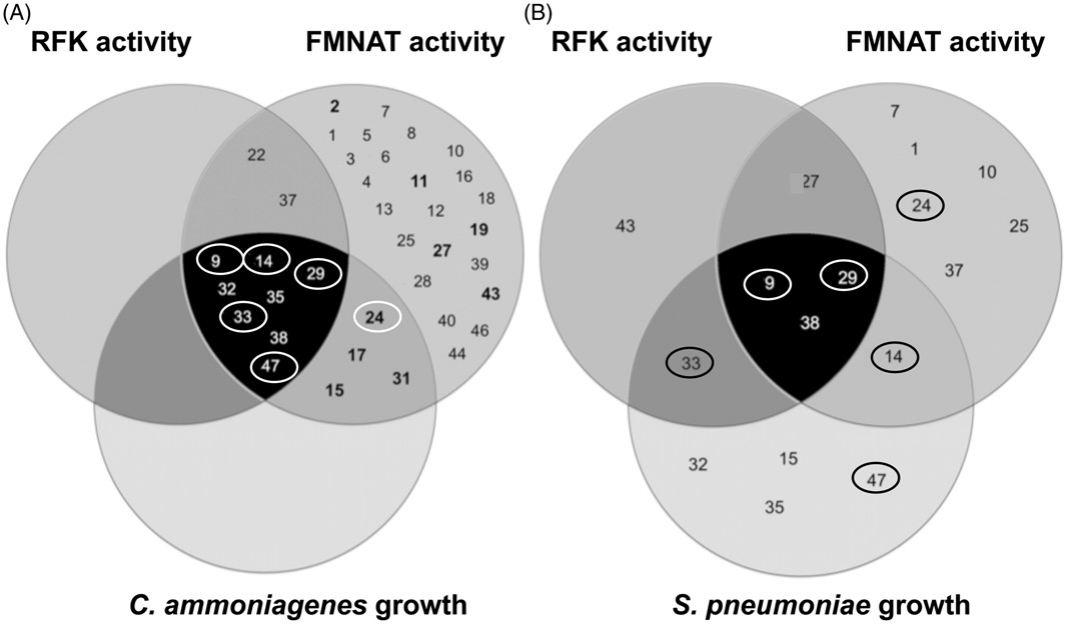
Venn diagrams for the HTS hits effects on *C. ammoniagenes* and *S. pneumoniae.* (A) HTS hits that inhibit the RFK (dark grey circle) and FMNAT (medium grey circle) activities of *Ca*FADS as well as the growth of *C. ammoniagenes* cells (pale grey circle). (B) HTS hits that inhibit the RFK (dark grey circle) and FMNAT (medium grey circle) activities of *Spn*FADS and the *S. pneumoniae* cellular growth (pale grey circle). In (A), The hits whose inhibition potency was experimentally assessed in this study (inhibit the FMNAT activity without affecting the RFK one) are highlighted in bold. The hits surrounded by a circle, both in (A) and (B), also inhibit the proliferation of *M. tuberculosis*.

To assess the effect of the HTS hits on the growth of different bacteria, we determined their MIC (Table 6). Bacterial cells of *C. ammoniagenes*, *M. tuberculosis* and *S. pneumoniae* were grown in the presence of increasing concentrations of the selected HTS hits. Among the 37 HTS hits, only twelve, six and nine compounds inhibited, respectively, the growth of *C. ammoniagenes*, *M. tuberculosis* and *S. pneumoniae*. Among these, only 9, benzethonium chloride (14), methyl-benzethonium chloride (29), alexidine dihydrochloride (33) and verteporfin (47) showed MIC values for *C. ammoniagenes* lower than 2 µM, while 17, 24, diethylstilbestrol (32) and dienestrol (35) show values between 2 and 16 µM (Table 6). Interestingly, the compounds that presented better properties against the *Ca*FADS FMNAT activity (24, 27 and 31) have a poor growth inhibitory effect on the bacterial cells (24 and 27 have MIC ≈ 32 and 64 µM, respectively, whereas the MIC of 31 was >64 µM). Nonetheless, it is worth to notice that 9, 14, 29, 33 and 47 show in common the considerabl ability to inhibit both of the *Ca*FADS enzymatic activities as well as to greatly affect the *C. ammoniagenes* growth (Table 1, Figure 7). Regarding *M. tuberculosis* growth, sulfasalazine (8), 9, 24 and 29 produced mild effects on cell growth, but only 14 and 33 showed MIC values below 8 µM. Table 6 summarises the effect of some selected compounds on *S. pneumoniae*, indicating that 9, 14, 29 and 33 inhibit moderately its growth.

**Table 6.** Minimal inhibitory concentration (MIC) of selected HTS hits against different microorganisms.

HTS hit	*C. ammoniagenes* (μM)	*M. tuberculosis* (μM)	*S. pneumoniae* (μM)
1	>64	>64	>64
*2*	>64	>64	>64
9	2	16	1-2
*11*	>64	>64	>64
14	0.25	8	2
*15*	32	>64	64
*17*	8	>64	>64
*24*	16–32	16–32	>64
*27*	64	>64	>64
29	0.125	16–32	1
*31*	32–64	>64	>64
32	8	>64	64
33	0.125	2–4	0.5
35	16–32	>64	64
37	>64	>64	>64
38	32	>64	16–32
47	2–4	8	4

The FMNAT hits are shown in italics. Compounds were assayed in the 0.125–64 μM range.

## Discussion

### Validation of the HTS protocol

Here we have designed and optimised an enzymatic activity-based HTS protocol to discover specific inhibitors for *Ca*FADS. In this protocol, the direct evaluation of the FMNAT enzymatic activity allows for the selection of species-specific inhibitors^38^^,^^39^. Our HTS protocol is simple, effective and consumes little protein (1.5 ng/compound against the ∼5 µg/compound required for some differential scanning fluorescence-based HTSs). Our protocol monitors directly the activity of the enzyme that we want to inhibit (Figure 1(A)), therefore, minimises the number of false positives and PAINS. In addition, because the chemicals in the library are approved drugs, their toxicity in mammalian cells is expected to be limited. The 37 HTS hits obtained (3.6% of the chemical library) were further assayed against the RFK and FMNAT activities of *Ca*FADS. Nine of the HTS hits (when assayed at 250 µM) almost completely inhibited the FMNAT activity without practically affecting the RFK one (Table 1). Because the FMNAT activity of *Ca*FADS appeared as a preferred target, due to the different active site regarding eukaryotic enzymes, we further assessed the inhibitory power of these FMNAT hits.

### Inhibitors targeting the FMNAT activity of CaFADS

We choose the three FMNAT hits that showed the lowest IC_50_ and residual activity (24, 27 and 31, 8% of the HTS hits) (Table 2) to further determine their binding affinities and inhibition mechanisms. Chicago Sky Blue 6B (CSB), here 24, is an allosteric inhibitor of the Macrophage Inhibitor Factor, and shows promising *in vivo*-effects for the treatment of spinal cord injury^40^. Additionally, 24 can act as an anticancer drug through the specific inhibition of Rad 51, as well as a potential resource for Alzheimer disease treatment by inhibiting the binding of β-amyloid to the prion protein^41^^,^^42^. Furthermore, the inhibition of vesicular glutamate transporters by 24 attenuates expressions of behavioural sensitization^43^. In our study, CSB is the most potent inhibitor of the *Ca*FADS FMNAT activity, as shown by its lowest values of residual activity and IC_50_ (Table 2), targeting the free enzyme as well as the FMN-protein and ATP-protein complexes (Table 3, Supplementary Figure SD2). Moreover, 24 binds to both enzyme modules being the binding to the FMNAT site highly favourable and enthalpically driven (Table 4, Figure 5). This is an advantage for an inhibitor, since high binding enthalpy denotes lots of specific interactions^44^.

Gossypol, here 27, was used some years ago in China as a masculine contraceptive^45^, however, its side effects stopped its pharmacological use. More recently, 27 has demonstrated anticancer effects through the inhibition of antiapoptotic proteins belonging to the Bcl-2 family and of molecules implicated in tumour progression^46^^,^^47^. Additionally, 27 inhibits the HIV-1 replication *in vitro*^48^. 27 is a potent inhibitor of the *Ca*FADS FMNAT activity, as shown by the low IC_50_ and residual FMNAT activity (Table 2), while does not affect the RFK one (Table 1, Figure 2). This compound competes with ATP for its binding at the FMNAT site (Table 3, Figures 4 and 6(C)). The lower *K*_d_ value for 27 when compared with that of FMN^29^, makes the inhibitor a preferred ligand for *Ca*FADS.

31, or flunixin meglumine, is a non-steroidal anti-inflammatory drug, analgesic and antipyretic extensively used in horses, pigs and cattle^49–51^. This fact guarantees that 31 can be used securely in mammals. In this study, 31 arises as a mixed inhibitor of the *Ca*FADS FMNAT activity. This is in agreement with our docking model and its small size, envisaging that binding of this compound might coexist with binding of substrates or products in non-competent conformations. Although 31 is the less potent inhibitor of the three here characterised (Table 2, Figure 3), its binding thermodynamic properties, together with its bio-security in mammals, reveals its potentiality as a drug precursor.

Our docking models supply additional details about the molecular inhibition mechanisms of 24, 27 and 31. Because 27 is a competitive inhibitor it occupies the active site substituting ATP, as demonstrated with the best docking poses (Figure 6(C)). However, the way in which 24 and 31 inhibit the FMNAT activity is not obvious. The 31 binding conformation (Figure 6(D)) could be affected by the ATP ligand, and, given the small size of 31, it could coexist with ATP in the active site, causing the previously described intricate mechanism. In presence of ATP, the 24 binding conformation should be different to the one of our FADS:24_1_ model, because ATP has to displace 24 from the binding site, given that 24 partially occupies it^24^. It is probable that with ATP in the active site, and taking into account the large size of this molecule, 24 only interacts with the active site via the N-terminal of the α6n helix (pink molecule in Figure 6(B)) and the flexible loop L4n that forms the external and entry part of the FMNAT substrates cavities^23^^,^^24^. In this way, the 24 sulphates would be able to coordinate the magnesium ion to potentially induce a change in the ATP phosphates orientation, negatively affecting their orientation for the FMNAT activity as well as the entry and exit of the reaction ligands.

### Inhibition of other bacterial FADS by CaFADS FMNAT hits

*S. pneumoniae* causes more than 25% of the cases of community-acquired pneumonia^52^, generating more deaths than any other vaccine-preventable bacterial disease. *M. tuberculosis* causes tuberculosis, the most common cause of death among infectious diseases^53^. *Spn*FADS has as similar native structure to *Ca*FADS (Supplementary Figure SD1), while the *Mt*FADS sequence shows 45% identity with *Ca*FADS and 59% of conservation^30^. Since the attempts to produce stable purified *Mt*FADS have so far failed, we considered *Ca*FADS a good model for *Mt*FADS, as reported for other proteins of these two genera^54^. Thus, we tested the effect of the *Ca*FADS HTS hits on the RFK and FMNAT activities of *Spn*FADS. We can find that only 30% of the HTS hits have inhibitory effects on *Spn*FADS, and the high values of residual activities and IC_50_ reveal that they are worse inhibitors for *Spn*FADS (Table 5). Nevertheless, among the HTS hits, 9, 27, 37 and 43 are interesting inhibitors of *Spn*FADS (Table 5). 27 and 43, which did not inhibit the *Ca*FADS RFK activity (Table 1), have an important effect on the *Spn*FADS one. 9, inhibits both *Spn*FADS activities (Table 5). This compound is a monocationic detergent that has been commonly used in cortisporin-TC ear drops to help penetration of active ingredients through cellular debris. Additionally, 9 inhibits vacuolar ATPase, showing cytotoxic effects at concentrations higher than 10 µM^55^. It is also an inhibitor of the RANK-L induced osteoclast formation^56^. 37, inhibited both *Ca*FADS activities, but only affects the FMNAT one in *Spn*FADS. 37 inhibits the HMG-CoA reductase, being used to treat hypercholesterolemia^57^. 37 has positive effects in myocardial fibrosis by favouring ACE2 expression, and also modestly inhibits replication of the hepatitis C virus^58^^,^^59^. 43 is used as analgesic (inhibits anandamide hydrolase in neurons) and as anti-inflammatory^60^^,^^61^, acting as a no selective cyclooxygenase inhibitor. Additionally, it inhibits NF-κβ in activated monocytes, being a promising drug for the treatment of rheumatoid arthritis^62^. Regarding the three FMNAT hits of *Ca*FADS: 24 has a poor inhibitory effect on *Spn*FADS (Table 5), which might be of interest as selective inhibitor; 27 inhibits both *Ca*FADS and *Spn*FADS activities (Tables 1 and 5), appearing as a broad inhibitor of FADS; and 31 has no effect on *Spn*FADS (Table 5), indicating the specificity of this compound for *Ca*FADS.

Recent studies^23^^,^^28^^,^^63–65^ revealed important differences in catalysis among bifunctional FADS, probably related to dissimilarities in the active site conformation during catalysis. This is consistent with the differential inhibitory mechanisms of the HTS hits against *Ca*FADS and *Spn*FADS, as observed in the present work. Such mechanistic variations could determine the binding or the inhibitory capability of the hits. These observations highlight the potentiality of our method to find selective drugs targeting a specific protein of a particular microorganism. The development of such species-selective drugs is of great importance for the treatment of infections by avoiding undesired side-effects on normal microbiota of the host^66^, and for minimising the selection of resistant bacterial strains.

### Antimicrobial activity of the CaFADS FMNAT hits

Finally, we also tested the inhibitory activity of the best FMNAT hits on the growth of *C. ammoniagenes*, *S. pneumoniae* and *M. tuberculosis* cultures. 24 shows moderate effects on the *C. ammoniagenes* and *M. tuberculosis* growth (Table 6, Figure 7). However, it does not inhibit the pneumococci growth. 27 and 31 do not inhibit the growth of *C. ammoniagenes*, *S. pneumoniae* or *M. tuberculosis* cells. This might be due to their inability to enter in the bacterial cell, or because efflux pumps eject them once in the bacterial cytoplasm. Tools to favour their bactericide effects can be obtained by deriving second generation hits, using vehiculization systems to move drugs across the membrane, or using efflux pumps inhibitors^67–69^. In this context, we remark that inhibition of FAD synthesis in *M. tuberculosis* could have an immediate impact in current antituberculosis drug discovery programmes. Benzothiazinones are antituberculosis compounds that block arabinan synthesis by targeting the flavoprotein decaprenylphosphoryl-β-D-ribose 2'-epimerase DprE1^70^. It is expected that the antituberculosis activity of benzothiazinones, which are currently in phase I clinical trials^4^, could be enhanced by FADS inhibition, in a synergistic manner. Among the other FMNAT hits, only 15, 17 and 31 show mild inhibitory activity on the growth of *C. ammoniagenes*, but do not have an effect on *S. pneumoniae* and *M. tuberculosis* cultures (Figure 7).

We find five HTS hits (9, 14, 29, 33 and 47) as strong inhibitors of the *C. ammoniagenes* growth (Table 6, Figure 7). These five HTS hits also inhibit the growth of *S. pneumoniae* (Table 6, Figure 7), while only the first four mildly affect *M. tuberculosis*. Noticeably, these five HTS hits are good inhibitors of both *Ca*FADS activities (Table 1, Figure 7). 9 and 29 also appear as potent inhibitors of the *Spn*FADS RFK and/or FMNAT activities. The effect of the other three compounds as *Spn*FADS inhibitors is milder, suggesting mechanisms that do not involve FADS in preventing cell proliferation. Overall, our results suggest that targeting both activities of bifunctional FADSs can be a strategy in the discovery of new antibacterial drugs. Thus, the non-selective antimicrobial properties of 9 and 29 seem interesting tools to be explored.

In this context, and despite all compounds in the library are approved by FAD and EMA, it remains for future studies to test the effect of the most promising compounds on the homologous human proteins and cells. In addition, future studies should also focus on the improvement of these compounds regarding potency, selectivity, pharmacokinetics and drug-likeness.

## Conclusions

The FMNAT activity of bifunctional FADS enzymes is a potential antimicrobial target for drug discovery. The transformation of FMN into FAD is performed by different catalytic mechanisms in prokaryotes and eukaryotes, and the FMN and FAD deficiency inactivates an important number of flavoproteins. In this work, we have optimised an activity-based HTS that can be used to discover new antibacterial drugs, targeting the RFK and/or FMNAT activities of bifunctional FADSs. Our method allows identifying bacterial FADS inhibitors with different levels of selectivity regarding the inhibition of bacterial growth. The method is fast, effective and requires small protein quantities. We have confirmed that bacterial FADS are promising species-selective drug targets. Among the 1240 compounds from the Prestwick Chemical Library^®^, 37 inhibited *Ca*FADS, and three were potent inhibitors of its FMNAT (but not of the RFK) activity. Two of these compounds were not species-selective because they also affected *Spn*FADS, but the third one, 31, was selective for *Ca*FADS versus *Spn*FADS. These three compounds are promising as non-selective or selective inhibitors at the enzyme level. However, they do not produce observable antimicrobial effects, suggesting that they do not reach inhibitory concentrations at the intracellular level, possibly due to a poor uptake, efficient efflux or *in vivo* fast degradation. Nevertheless, some HTS hits show good antimicrobial properties, probably due to the inhibition of both RFK and FMNAT activities of FADS.

## Supplementary Material

IENZ_1411910_Supplementary_Material.pdf

## References

[1] Spellberg B, Powers JH, Brass EP, et al Trends in antimicrobial drug development: implications for the future. Clin Infect Dis 2004;38:1279–86. 15127341 10.1086/420937

[2] Conly J, Johnston B. Where are all the new antibiotics? The new antibiotic paradox. Can J Infect Dis Med Microbiol 2005;16:159–60. 18159536 10.1155/2005/892058 PMC2095020

[3] Walsh CT, Wencewicz TA. Prospects for new antibiotics: a molecule-centered perspective. J Antibiot (Tokyo) 2014;67:7–22. 23756684 10.1038/ja.2013.49

[4] WHO Antibacterial agents in clinical development: an analysis of the antibacterial clinical development pipeline, including tuberculosis. WHO/EMP/IAU/2017.11 ed. World Health Organization: Geneva; 2017.

[5] Brown ED, Wright GD. Antibacterial drug discovery in the resistance era. Nature 2016;529:336–43. 26791724 10.1038/nature17042

[6] Lienhart WD, Gudipati V, Macheroux P. The human flavoproteome. Arch Biochem Biophys 2013;535:150–62. 23500531 10.1016/j.abb.2013.02.015 PMC3684772

[7] Gudipati V, Koch K, Lienhart WD, Macheroux P. The flavoproteome of the yeast *Saccharomyces cerevisiae*. Biochim Biophys Acta 2014;1844:535–44. 24373875 10.1016/j.bbapap.2013.12.015 PMC3991850

[8] Barile M, Giancaspero TA, Leone P, et al Riboflavin transport and metabolism in humans. J Inherit Metab Dis 2016;39:545–57. 27271694 10.1007/s10545-016-9950-0

[9] Gross E, Kastner DB, Kaiser CA, Fass D. Structure of Ero1p, source of disulfide bonds for oxidative protein folding in the cell. Cell 2004;117:601–10. 15163408 10.1016/s0092-8674(04)00418-0

[10] Parsons HG, Dias VC. Intramitochondrial fatty acid metabolism: riboflavin deficiency and energy production. Biochem Cell Biol 1991;69:490–7. 1793560 10.1139/o91-073

[11] Myllykallio H, Lipowski G, Leduc D, et al An alternative flavin-dependent mechanism for thymidylate synthesis. Science 2002;297:105–7. 12029065 10.1126/science.1072113

[12] Susin SA, Lorenzo HK, Zamzami N, et al Molecular characterization of mitochondrial apoptosis-inducing factor. Nature 1999;397:441–6. 9989411 10.1038/17135

[13] Sassetti CM, Boyd DH, Rubin EJ. Genes required for mycobacterial growth defined by high density mutagenesis. Mol Microbiol 2003;48:77–84. 12657046 10.1046/j.1365-2958.2003.03425.x

[14] Griffin JE, Gawronski JD, Dejesus MA, et al High-resolution phenotypic profiling defines genes essential for mycobacterial growth and cholesterol catabolism. PLoS Pathog 2011;7:e1002251. 21980284 10.1371/journal.ppat.1002251 PMC3182942

[15] Serrano A, Ferreira P, Martínez-Júlvez M, Medina M. The prokaryotic FAD synthetase family: a potential drug target. Curr Pharm Des 2013;19:2637–48. 23116401 10.2174/1381612811319140013

[16] Barile M, Passarella S, Bertoldi A, Quagliariello E. Flavin adenine dinucleotide synthesis in isolated rat liver mitochondria caused by imported flavin mononucleotide. Arch Biochem Biophys 1993;305:442–7. 8373181 10.1006/abbi.1993.1444

[17] Serrano A, Frago S, Velázquez-Campoy A, Medina M. Role of key residues at the flavin mononucleotide (FMN):adenylyltransferase catalytic site of the bifunctional riboflavin kinase/flavin adenine dinucleotide (FAD) synthetase from *Corynebacterium ammoniagenes*. Int J Mol Sci 2012;13:14492–517. 23203077 10.3390/ijms131114492 PMC3509593

[18] Efimov I, Kuusk V, Zhang X, McIntire WS. Proposed steady-state kinetic mechanism for *Corynebacterium ammoniagenes* FAD synthetase produced by *Escherichia coli*. Biochemistry 1998;37:9716–23. 9657684 10.1021/bi972817j

[19] Mack M, van Loon AP, Hohmann HP. Regulation of riboflavin biosynthesis in *Bacillus subtilis* is affected by the activity of the flavokinase/flavin adenine dinucleotide synthetase encoded by ribC. J Bacteriol 1998;180:950–5. 9473052 10.1128/jb.180.4.950-955.1998 PMC106977

[20] Herguedas B, Martínez-Júlvez M, Frago S, et al Crystallization and preliminary X-ray diffraction studies of FAD synthetase from *Corynebacterium ammoniagenes*. Acta Crystallogr Sect F Struct Biol Cryst Commun 2009;65:1285–8. 10.1107/S1744309109044789 PMC2802882 20054130

[21] Wang W, Kim R, Yokota H, Kim SH. Crystal structure of flavin binding to FAD synthetase of *Thermotoga maritima*. Proteins 2005;58:246–8. 15468322 10.1002/prot.20207

[22] Wang W, Kim R, Jancarik J, et al Crystal structure of a flavin-binding protein from *Thermotoga maritima*. Proteins 2003;52:633–5. 12910462 10.1002/prot.10353

[23] Sebastián M, Lira-Navarrete E, Serrano A, et al The FAD synthetase from the human pathogen *Streptococcus pneumoniae*: a bifunctional enzyme exhibiting activity-dependent redox requirements. Sci Rep 2017;7:7609. 28790457 10.1038/s41598-017-07716-5 PMC5548840

[24] Herguedas B, Martinez-Julvez M, Frago S, et al Oligomeric state in the crystal structure of modular FAD synthetase provides insights into its sequential catalysis in prokaryotes. J Mol Biol 2010;400:218–30. 20471397 10.1016/j.jmb.2010.05.018

[25] Giancaspero TA, Galluccio M, Miccolis A, et al Human FAD synthase is a bi-functional enzyme with a FAD hydrolase activity in the molybdopterin binding domain. Biochem Biophys Res Commun 2015;465:443–9. 26277395 10.1016/j.bbrc.2015.08.035

[26] Serrano A, Sebastian M, Arilla-Luna S, et al Quaternary organization in a bifunctional prokaryotic FAD synthetase: involvement of an arginine at its adenylyltransferase module on the riboflavin kinase activity. Biochim Biophys Acta 2015;1854:897–906. 10.1016/j.bbapap.2015.03.005 25801930

[27] Serrano A, Frago S, Herguedas B, et al Key residues at the riboflavin kinase catalytic site of the bifunctional riboflavin kinase/FMN adenylyltransferase from Corynebacterium ammoniagenes. Cell Biochem Biophys 2013;65:57–68. 22892871 10.1007/s12013-012-9403-9

[28] Herguedas B, Lans I, Sebastián M, et al Structural insights into the synthesis of FMN in prokaryotic organisms. Acta Crystallogr D Biol Crystallogr 2015;71:2526–42. 26627660 10.1107/S1399004715019641

[29] Frago S, Velázquez-Campoy A, Medina M. The puzzle of ligand binding to *Corynebacterium ammoniagenes* FAD synthetase. J Biol Chem 2009;284:6610–19. 19136717 10.1074/jbc.M808142200 PMC2652324

[30] Frago S, Martínez-Júlvez M, Serrano A, Medina M. Structural analysis of FAD synthetase from *Corynebacterium ammoniagenes*. BMC Microbiol 2008;8:160. 18811972 10.1186/1471-2180-8-160 PMC2573891

[31] Lagorce D, Oliveira N, Miteva MA, Villoutreix BO. Pan-assay interference compounds (PAINS) that may not be too painful for chemical biology projects. Drug Discov Today 2017;22:1131–3. 28676405 10.1016/j.drudis.2017.05.017

[32] Cosconati S, Forli S, Perryman AL, et al Virtual screening with AutoDock: theory and practice. Expert Opin Drug Discov 2010;5:597–607. 21532931 10.1517/17460441.2010.484460 PMC3083070

[33] Morris GM, Huey R, Lindstrom W, et al AutoDock4 and AutoDockTools4: automated docking with selective receptor flexibility. J Comput Chem 2009;30:2785–91. 19399780 10.1002/jcc.21256 PMC2760638

[34] Forli S, Olson AJ. A force field with discrete displaceable waters and desolvation entropy for hydrated ligand docking. J Med Chem 2012;55:623–38. 22148468 10.1021/jm2005145 PMC3319101

[35] Frisch MJ, Trucks GW, Schlegel HB, et al Gaussian 09. Wallingford, CT: Gaussian Inc.; 2016.

[36] Palomino JC, Martin A, Camacho M, et al Resazurin microtiter assay plate: simple and inexpensive method for detection of drug resistance in Mycobacterium tuberculosis. Antimicrob Agents Chemother 2002;46:2720–2. 12121966 10.1128/AAC.46.8.2720-2722.2002 PMC127336

[37] Olsson TS, Williams MA, Pitt WR, Ladbury JE. The thermodynamics of protein-ligand interaction and solvation: insights for ligand design. J Mol Biol 2008;384:1002–17. 18930735 10.1016/j.jmb.2008.09.073

[38] Deu E, Yang Z, Wang F, et al Use of activity-based probes to develop high throughput screening assays that can be performed in complex cell extracts. PLoS One 2010;5:e11985. 20700487 10.1371/journal.pone.0011985 PMC2916841

[39] Zhang G. Protease assays. Available from: http://www.ncbi.nlm.nih.gov/books/NBK92006/

[40] Saxena T, Loomis KH, Pai SB, et al Nanocarrier-mediated inhibition of macrophage migration inhibitory factor attenuates secondary injury after spinal cord injury. ACS Nano 2015;9:1492–505. 25587936 10.1021/nn505980z

[41] Normand A, Rivière E, Renodon-Cornière A. Identification and characterization of human Rad51 inhibitors by screening of an existing drug library. Biochem Pharmacol 2014;91:293–300. 25124703 10.1016/j.bcp.2014.07.033

[42] Risse E, Nicoll AJ, Taylor WA, et al Identification of a compound that disrupts binding of Amyloid-β to the prion protein using a novel fluorescence-based assay. J Biol Chem 2015;290:17020–8. 25995455 10.1074/jbc.M115.637124 PMC4505445

[43] He Z, Yan L, Yong Z, et al Chicago sky blue 6B, a vesicular glutamate transporters inhibitor, attenuates methamphetamine-induced hyperactivity and behavioral sensitization in mice. Behav Brain Res 2013;239:172–6. 23159705 10.1016/j.bbr.2012.11.003

[44] Klebe G. Applying thermodynamic profiling in lead finding and optimization. Nat Rev Drug Discov 2015;14:95–110. 25614222 10.1038/nrd4486

[45] Soufir JC. Hormonal, chemical and thermal inhibition of spermatogenesis: contribution of French teams to international data with the aim of developing male contraception in France. Basic Clin Androl 2017;27:3. 28101363 10.1186/s12610-016-0047-2 PMC5237323

[46] Ferdek PE, Jakubowska MA, Nicolaou P, et al BH3 mimetic-elicited Ca(2+) signals in pancreatic acinar cells are dependent on Bax and can be reduced by Ca(2+)-like peptides. Cell Death Dis 2017;8:e2640. 28252652 10.1038/cddis.2017.41 PMC5386550

[47] Xiong J, Li J, Yang Q, et al Gossypol has anti-cancer effects by dual-targeting MDM2 and VEGF in human breast cancer. Breast Cancer Res 2017;19:27 28274247 10.1186/s13058-017-0818-5 PMC5343402

[48] Polsky B, Segal SJ, Baron PA, et al Inactivation of human immunodeficiency virus in vitro by gossypol. Contraception 1989;39:579–87. 2473865 10.1016/0010-7824(89)90034-6

[49] Newby NC, Leslie KE, Dingwell HD, et al The effects of periparturient administration of flunixin meglumine on the health and production of dairy cattle. J Dairy Sci 2017;100:582–7. 27865498 10.3168/jds.2016-11747

[50] Kleinhenz MD, Van Engen NK, Gorden PJ, et al The pharmacokinetics of transdermal flunixin meglumine in Holstein calves. J Vet Pharmacol Ther 2016;39:612–15. 27121728 10.1111/jvp.12314

[51] Burkett BN, Thomason JM, Hurdle HM, et al Effects of firocoxib, flunixin meglumine, and phenylbutazone on platelet function and thromboxane synthesis in healthy horses. Vet Surg 2016;45:1087–94. 27731498 10.1111/vsu.12567

[52] Torres A, Blasi F, Peetermans WE, et al The aetiology and antibiotic management of community-acquired pneumonia in adults in Europe: a literature review. Eur J Clin Microbiol Infect Dis 2014;33:1065–79. 24532008 10.1007/s10096-014-2067-1 PMC4042014

[53] WHO Global Tuberculosis Report. Geneva: WHO; 2016.

[54] Seidel M, Alderwick LJ, Sahm H, et al Topology and mutational analysis of the single Emb arabinofuranosyltransferase of *Corynebacterium glutamicum* as a model of Emb proteins of *Mycobacterium tuberculosis*. Glycobiology 2007;17:210–19. 17088267 10.1093/glycob/cwl066

[55] Chan CY, Prudom C, Raines SM, et al Inhibitors of V-ATPase proton transport reveal uncoupling functions of tether linking cytosolic and membrane domains of V0 subunit a (Vph1p). J Biol Chem 2012;287:10236–50. 22215674 10.1074/jbc.M111.321133 PMC3323027

[56] Zhu X, Gao JJ, Landao-Bassonga E, et al Thonzonium bromide inhibits RANKL-induced osteoclast formation and bone resorption in vitro and prevents LPS-induced bone loss in vivo. Biochem Pharmacol 2016;104:118–30. 26906912 10.1016/j.bcp.2016.02.013

[57] Weng TC, Yang YH, Lin SJ, Tai SH. A systematic review and meta-analysis on the therapeutic equivalence of statins. J Clin Pharm Ther 2010;35:139–51. 20456733 10.1111/j.1365-2710.2009.01085.x

[58] Shin YH, Min JJ, Lee JH, et al The effect of fluvastatin on cardiac fibrosis and angiotensin-converting enzyme-2 expression in glucose-controlled diabetic rat hearts. Heart Vessels 2017;32:618–27. 10.1007/s00380-016-0936-5 28013371

[59] Bader T, Fazili J, Madhoun M, et al Fluvastatin inhibits hepatitis C replication in humans. Am J Gastroenterol 2008;103:1383–9. 18410471 10.1111/j.1572-0241.2008.01876.x

[60] Dallegri F, Bertolotto M, Ottonello L. A review of the emerging profile of the anti-inflammatory drug oxaprozin. Expert Opin Pharmacother 2005;6:777–85. 15934904 10.1517/14656566.6.5.777

[61] Kara IM, Polat S, Inci MF, et al Analgesic and anti-inflammatory effects of oxaprozin and naproxen sodium after removal of impacted lower third molars: a randomized, double-blind, placebo-controlled crossover study. J Oral Maxillofac Surg 2010;68:1018–24. 20206429 10.1016/j.joms.2009.09.094

[62] Montecucco F, Bertolotto M, Ottonello L, et al Oxaprozin-induced apoptosis on CD40 ligand-treated human primary monocytes is associated with the modulation of defined intracellular pathways. J Biomed Biotechnol 2009;2009:478785. 19672323 10.1155/2009/478785 PMC2723963

[63] Grill S, Busenbender S, Pfeiffer M, et al The bifunctional flavokinase/flavin adenine dinucleotide synthetase from *Streptomyces davawensis* produces inactive flavin cofactors and is not involved in resistance to the antibiotic roseoflavin. J Bacteriol 2008;190:1546–53. 18156273 10.1128/JB.01586-07 PMC2258686

[64] Matern A, Pedrolli D, Groszhennig S, et al Uptake and metabolism of antibiotics roseoflavin and 8-demethyl-8-aminoriboflavin in riboflavin-auxotrophic listeria monocytogenes. J Bacteriol 2016;198:3233–43. 27672192 10.1128/JB.00388-16 PMC5105903

[65] Marcuello C, Arilla-Luna S, Medina M, Lostao A. Detection of a quaternary organization into dimer of trimers of Corynebacterium ammoniagenes FAD synthetase at the single-molecule level and at the in cell level. Biochim Biophys Acta 2013;1834:665–76. 23291469 10.1016/j.bbapap.2012.12.013

[66] Lewis K. Platforms for antibiotic discovery. Nat Rev Drug Discov 2013;12:371–87. 23629505 10.1038/nrd3975

[67] Stermitz FR, Lorenz P, Tawara JN, et al Synergy in a medicinal plant: antimicrobial action of berberine potentiated by 5'-methoxyhydnocarpin, a multidrug pump inhibitor. Proc Natl Acad Sci USA 2000;97:1433–7. 10677479 10.1073/pnas.030540597 PMC26451

[68] Rodrigues L, Parish T, Balganesh M, Ainsa JA. Antituberculosis drugs: reducing efflux = increasing activity. Drug Discov Today 2017;22:592–99. 10.1016/j.drudis.2017.01.002 28089787

[69] Ladbury JE, Klebe G, Freire E. Adding calorimetric data to decision making in lead discovery: a hot tip. Nat Rev Drug Discov 2010;9:23–7. 19960014 10.1038/nrd3054

[70] Makarov V, Manina G, Mikusova K, et al Benzothiazinones kill *Mycobacterium tuberculosis* by blocking Arabinan synthesis. Science 2009;324:801–4. 19299584 10.1126/science.1171583 PMC3128490

