# Supplementary material for: Discovery of antimicrobial compounds targeting bacterial type FAD synthetases
Source: J Enzyme Inhib Med Chem. 2017 Dec 19;33(1):241–54. doi: 10.1080/14756366.2017.1411910 (PMC7012052; doi:10.1080/14756366.2017.1411910)
Supplement: IENZ_1411910_Supplementary_Material.pdf [file IENZ_A_1411910_SM9595.pdf]

## Supplementary Charts

**Chart SD1.** Structures of the 37 compounds selected as HTS hits for *Ca*FADS.

| Compound number | Name | CAS number | Structure |
|---|---|---|---|
| 1 | Diclofenac sodium | 15307-29-6 | 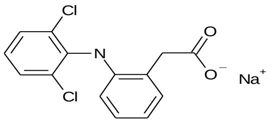 |
| 3 | Phenazopyridine hydrochloride | 136-40-3 | 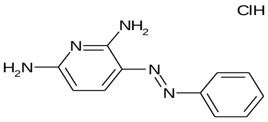 |
| 4 | Flufenamic acid | 530-78-9 | 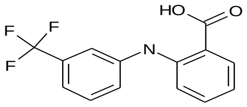 |
| 5 | Aceclofenac | 89796-99-6 | 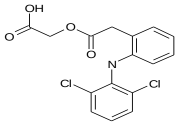 |
| 6 | Dipyridamole | 58-32-2 | 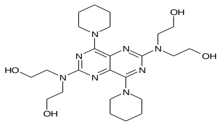 |
| 7 | Myricetin | 529-44-2 | 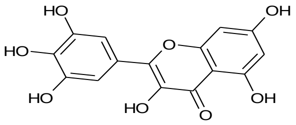 |
| 8 | Sulfasalazine | 599-79-1 | 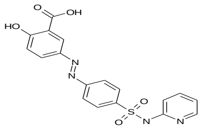 |
| 9 | Thonzonium bromide | 553-08-2 | 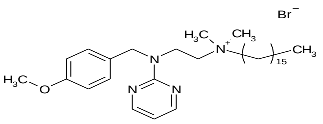 |
| 10 | Apigenin | 520-36-5 | 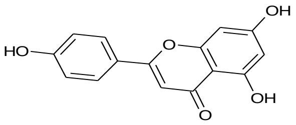 |
| 12 | Mefenamic acid | 61-68-7 | 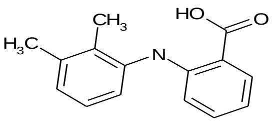 |

| Compound number | Name | CAS number | **Structure** |
|---|---|---|---|
| 13 | Diflunisal | 22494-42-4 | 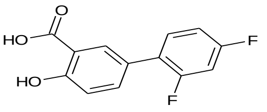 |
| 14 | Benzethonium chloride | 121-54-0 | 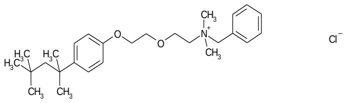 |
| 16 | Meclofenamic acid sodium salt monohydrate | 6385-02-0 | 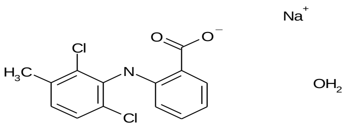 |
| 18 | Clomiphene citrate (Z,E) | 50-41-9 | 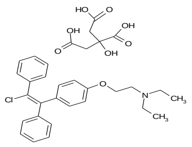 |
| 22 | Butoconazole nitrate | 64872-77-1 | 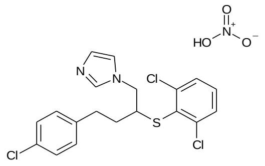 |
| 25 | Quercetine dihydrate | 6151-25-3 | 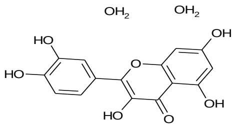 |
| 28 | Karakoline | 39089-30-0 | 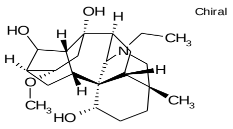 |
| 29 | Methyl-benzethonium chloride | 25155-18-4 | 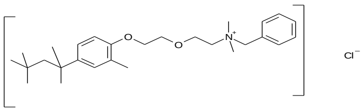 |
| 32 | Diethylstilbestrol | 56-53-1 | 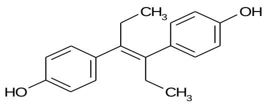 |
| 33 | Alexidine dihydrochloride | 1715-30-6 | 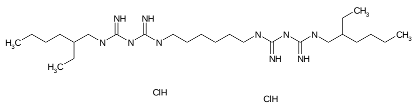 |

| Compound number | Name | CAS number | Structure |
|---|---|---|---|
| 35 | Dienestrol | 84-17-3 | 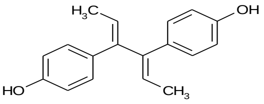 |
| 37 | Fluvastatin sodium salt | 93957-55-2 | 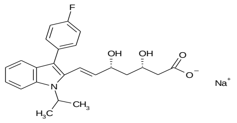 |
| 38 | Raloxifene hydrochloride | 82640-04-8 | 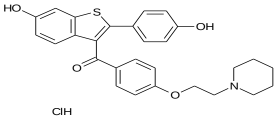 |
| 39 | Luteolin | 491-70-3 | 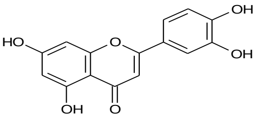 |
| 40 | (+)-Isoproterenol (+)-bitartrate salt | 54750-10-6 | 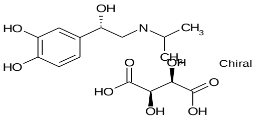 |
| 44 | (-)-Eseroline fumarate salt | 70310-73-5 | 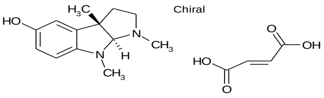 |
| 46 | Kaempferol | 520-18-3 | 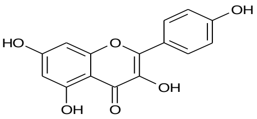 |
| 47 | Verteporfin | 129497-78-5 | 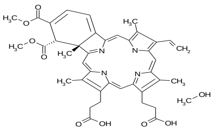 |

## Supplementary Figures

**Figure SD1**. Similarity within members of the bacterial bifunctional FADS family. (A) Sequence alignment of *Ca*FADS (Q59263), *Spn*FADS (A0A0H2UPY5) and *Mt*FADS (NP_217302.1) obtained using CLUSTAL W (http://www.ebi.ac.uk/clustalw/) with default parameters. The consensus sequence is indicated on the top. Equivalent residues at each position show same color. Deep and light blue upper bars indicate regions for the FMNAT and RFK modules, respectively. (B) Structures of *Ca*FADS (PDB 2X0K), with the FMNAT and RFK modules in blue and light blue respectively, and of *Spn*FADS (PDB 3OP1), with the FMNAT and RFK modules in pink and light pink respectively. The reaction catalyzed by each module is next to the corresponding module. On the right it is shown the structural alignment showing an r.m.s.d. of 1.63 Å for 247 Cα atoms. Structural figures were produced with PyMol (Delano 2002).

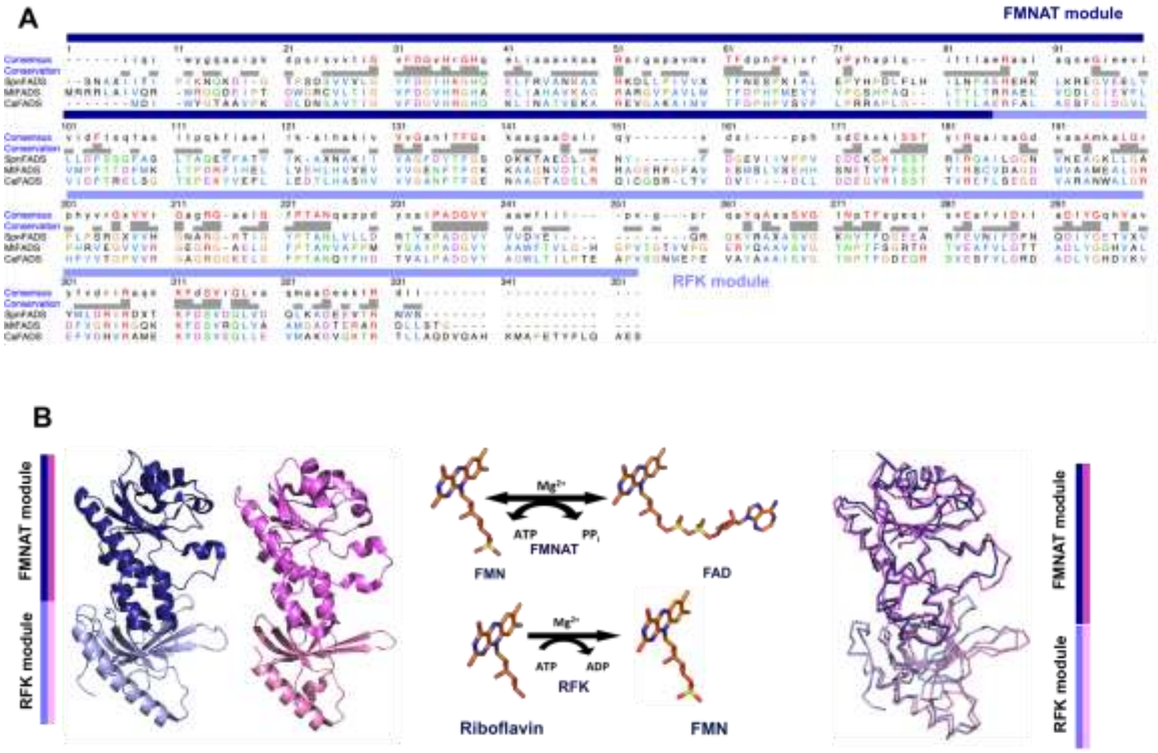

**Figure SD2.** Hit 24 as inhibitor of the FMNAT activity of *Ca*FADS. Michaelis-Menten plots at different concentrations of 24 and saturation (A) of ATP and (B) of FMN. Lineaweaver-Burk representations with global fit (C) to non-competitive inhibition model when ATP saturating and (D) to competitive inhibition model when FMN saturating. Reaction rates obtained in 20 mM PIPES, pH 7.0, 10 mM $MgCl_2$, 2.5 % DMSO, at 25 ºC, with 15 µM FMN and 10-450 µM ATP (FMN saturating) or with 350 µM ATP and 0.5-20 µM FMN (ATP saturating) (n=3, mean ± SD).

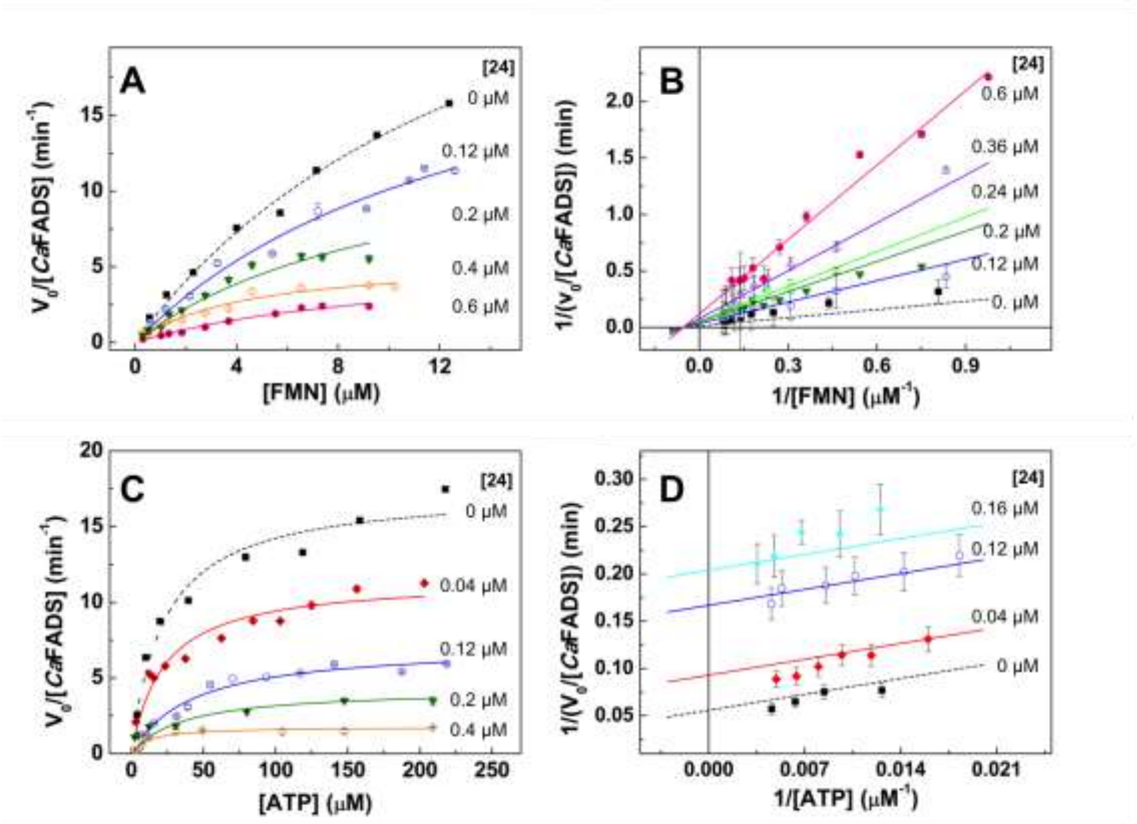

**Figure SD3**. Hit 31 as inhibitor of the FMNAT activity of *Ca*FADS. Michaelis-Menten plots at different concentrations of 31 and saturation (A) of ATP and (B) of FMN. (C) Lineaweaver-Burk representations with global fit to non-competitive inhibition model when ATP saturating, and (D) to competitive inhibition model when FMN saturating. Reaction rates obtained in 20 mM PIPES, pH 7.0, 10 mM $MgCl_2$, 2.5% DMSO, at 25 ºC, with 15 µM FMN and 10-450 µM ATP (FMN saturating) or with 350 µM ATP and 0.5-20 µM FMN (ATP saturating) (n=3, mean ± SD).

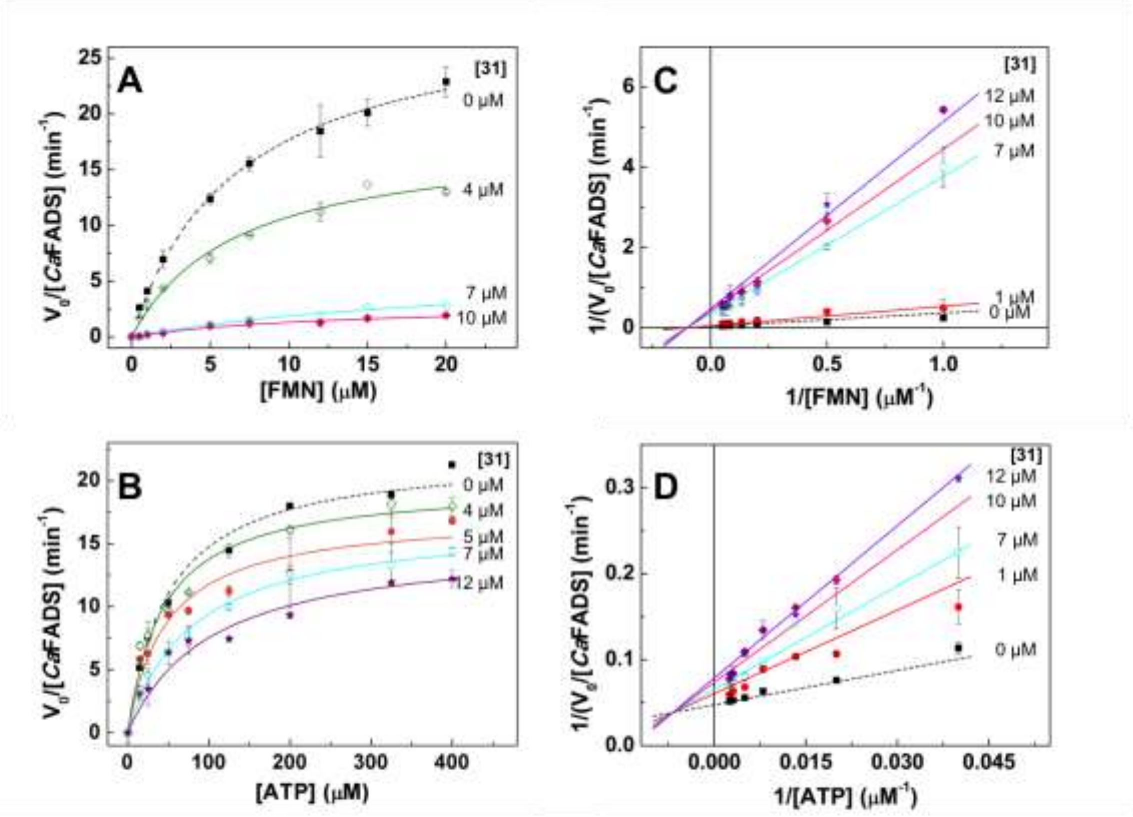

**Figure SD4**. Results from the docking analysis of the best five poses of the inhibitors (A) 24, (B) 27 (the five with almost identical orientation) and (C) 31 at the *Ca*FADS FMNAT site.  Inhibitors, FMNAT module and RFK module are showed in purple, gray and green, respectively. The poses for 24 and 27 correspond to the docking performed for the first molecular binding at the FMNAT module ($24_1$ and $27_1$).

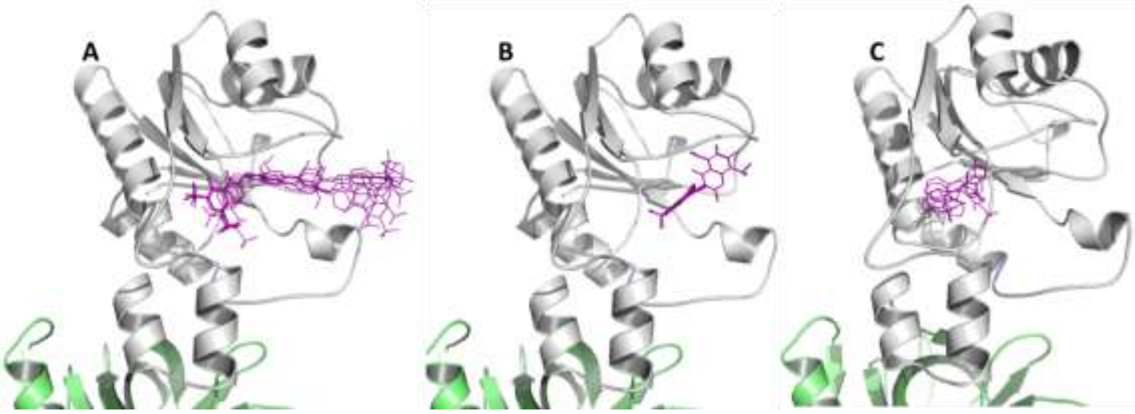

## Supporting Tables

**Table SP1**. Apparent kinetic parameters for the FMNAT activity of *Ca*FADS at different concentrations of the 24, 27 and 31 inhibitors. Data obtained at 25ºC in 20 mM PIPES, pH 7.0, 10 mM $MgCl_2$, 2.5% DMSO.

| | Saturating ATP | | | Saturating FMN | |
|---|---|---|---|---|---|
| [**24**] (μM) | $k_{cat}$ ($min^{-1}$) | $K_m$ (μM) | [**24**] (μM) | $k_{cat}$ ($min^{-1}$) | $K_m$ (μM) |
| 0 | 34 ± 3.8 | 16.3 ± 3.2 | 0 | 22.4 ± 0.7 | 29.4 ± 4.9 |
| 0.12 | 24.3 ± 4.1 [a] | 14.0 ± 3.9 | 0.04 | 22.5 ± 4.8 | 11.5 ± 0.7 [a] |
| 0.2 | 9.8 ± 1.3 [a] | 10.6 ± 2.4 | 0.12 | 7.2 ± 0.4 [a] | 42.1 ± 3.9 [a] |
| 0.4 | 5.6 ± 0.6 [a] | 4.3 ± 1.0 [a] | 0.2 | 4.3 ± 0.4 [a] | 35.8 ± 3.2 |
| 0.6 | 5.7 ± 1.3 [a] | 11.1 ± 3.9 | 0.4 | 1.8 ± 0.1 [a] | 12.0 ± 3.6 [a] |

| | Saturating ATP | | | Saturating FMN | |
|---|---|---|---|---|---|
| [**27**] (μM) | $k_{cat}$ ($min^{-1}$) | $K_m$ (μM) | [**27**] (μM) | $k_{cat}$ ($min^{-1}$) | $K_m$ (μM) |
| 0 | 34 ± 3.8 [a] | 16.3 ± 3.2 | 0 | 22.4 ± 0.7 | 29.4 ± 4.9 |
| 0.05 | 21.6 ± 2.9 [a] | 10.1 ± 2.9 | 0.1 | 22.8 ± 1.3 | 59.7 ± 5.7 [a] |
| 0.1 | 16.1 ± 1.7 [a] | 7.7 ± 2.1 | 0.2 | 22.2 ± 1.4 | 48.2 ± 5.6 [a] |
| 0.2 | 15.1 ± 1.5 [a] | 13.5 ± 2.6 | 0.4 | 18.1 ± 1.6 | 51.8 ± 5.5 [a] |
| 0.75 | 10.0 ± 0.8 [a] | 12.8 ± 2.1 | 0.75 | 17.1 ± 3.3 | 51.5 ± 4.9 [a] |
| 1 | 6.5 ± 0.2 [a] | 15.0 ± 2.0 | 1 | 12.5 ± 0.5 [a] | 40.9 ± 5.7 |

| | Saturating ATP | | | Saturating FMN | |
|---|---|---|---|---|---|
| [**31**] (μM) | $k_{cat}$ ($min^{-1}$) | $K_m$ (μM) | [**31**] (μM) | $k_{cat}$ ($min^{-1}$) | $K_m$ (μM) |
| 0 | 34 ± 3.8 | 16.3 ± 3.2 | 0 | 22.4 ± 0.7 | 29.4 ± 4.9 |
| 1 | 24.6 ± 2.9 [a] | 13.3 ± 3.1 | 4 | 19.7 ± 1.0 [a] | 43.3 ± 7.9 |
| 4 | 18.1 ± 1.4 [a] | 6.8 ± 1.3 | 5 | 17.3 ± 1.1 [a] | 47.5 ± 5.7 [a] |
| 5 | 6.4 ± 1.2 [a] | 24.4 ± 7.0 | 7 | 16.9 ± 0.6 [a] | 79.1 ± 8.7 [a] |
| 7 | 3.0 ± 0.4 [a] | 13.3 ± 3.6 | 10 | 15.3 ± 1.3 [a] | 102.9 ± 24.2 [a] |
| 10 | 3.0 ± 0.4 [a] | 12.3 ± 3.6 | 12 | 13.8 ± 1.0 [a] | 92.1 ± 14.9 [a] |

[a] Values showing statistically significant differences, $p < 0.002$, from the values in the absence of compounds, as determined by the one-way ANOVA test (n=3, confidence interval 95%).

Delano, W. L. (2002). "They PyMOL molecular graphics system." DeLano Scientific, San Carlos, CA, USA: http://www.pymol.org.
